# Integrative taxonomy of the genus *Coridius* Illiger, 1807 (Hemiptera: Heteroptera: Dinidoridae) reveals hidden diversity and three new species from North-East India

**DOI:** 10.1371/journal.pone.0298176

**Published:** 2024-07-31

**Authors:** Swapnil S. Boyane, Sandeep Sen, Dharma Rajan Priyadarsanan, Pavan Kumar Thunga, Nikhil U. Joshi, Hemant V. Ghate

**Affiliations:** 1 Ashoka Trust for Research in Ecology and the Environment (ATREE), Srirampura, Bangalore, India; 2 Mountain Science Center, University of Tsukuba, Nagano, Japan; 3 Post Graduate Research Centre, Department of Zoology, Modern College of Arts Commerce and Science, Pune, Maharashtra, India; University of Basrah, IRAQ

## Abstract

The genus *Coridius* Illiger, 1807 (Heteroptera: Dinidoridae) comprises a group of phytophagous terrestrial bugs consisting of 36 species distributed in the Afrotropical and Indo-Malayan regions. In several communities in northeastern India, insects are recognised as a delicacy, medicine, and a nutritional supplement, with *Coridius* being a popular delicacy. However, *Coridius* has received little taxonomic attention to date due to large intraspecific variations, inadequate taxonomic treatments, and the rarity of many species. To address this gap, an integrative taxonomy of the genus was performed. Two mitochondrial genes, viz., cytochrome oxidase subunit 1 (COI) and 16S rRNA, were sequenced to reconstruct the phylogenetic relationships within *Coridius*. We performed both maximum likelihood (ML) and Bayesian inference (BI) to develop a species tree, followed by the Bayesian implementation of the Poisson tree process (bPTP) and Assemble Species by Automatic Partitioning (ASAP) as an additional test to assess species boundaries and delimit operational taxonomic units. A linear discriminant analysis (LDA) of four key morphological characters was then performed to identify species groups. Overall, our analysis supported the establishment of three new species: *Coridius adii* **sp. nov.,** *Coridius esculentus* **sp. nov.,** and *Coridius insperatus* **sp. nov.,** and revealed six distinct lineages within *Coridius chinensis* (Dallas, 1851). Linear discriminant analysis of morphological characters indicated the clustering of eight species. The species status of *Coridius nigriventris* (Westwood, 1837) **stat. rev**, formerly synonymized under *Coridius nepalensis* (Westwood, 1837), is reinstated in this study. Further, we revised the genus *Coridius* from India and rediscovered *Coridius assamensis* (Distant, 1902) and *Coridius fuscus* (Westwood, 1837) after 100 years.

## Introduction

Species serve as the fundamental units of biodiversity [1], and reliable species delimitation and classification can enhance inferences in biosystematics and various other scientific fields, including biogeography, epidemiology, and conservation biology [2, 3]. However, accurate species delimitation often poses several conceptual and empirical challenges to biologists, which are further augmented while dealing with i) cryptic species [4], ii) lineages exhibiting shallow divergence [5], and iii) taxonomically complex systems [6]. Consequently, setting criteria for species delimitation has invoked a series of debates among systematic biologists in recent decades [7, 8]. A well-established species delineation criterion should be repeatable, provide independent assessments of morphologically defined lineages, and uncover previously unknown species [9]. Monophyly is one accepted criterion for supporting separate species [10]. However, taxonomic issues may arise when molecular phylogenies and morphological classifications are incongruent. In such cases, combining information from multiple yet conventionally disparate datasets and methodological approaches has proven useful in delineating species boundaries with better accuracy [11, 12].

Integrative taxonomy, which combines diverse datasets such as morphological, ecological, and molecular data (nuclear and mitochondrial DNA), has gained significant attention among biologists [13, 14]. This approach serves as a powerful tool for delineating species boundaries and offers a practical solution to the “taxonomic impediment,” a major obstacle in biodiversity research [15–17]. It allows researchers to harness the combined strength of various data types, including genetics, morphology, biochemical, and ecological information, to more accurately infer species boundaries [3, 18]. This approach is particularly relevant when inferences based on morphology fail, especially when dealing with lineages that contain species complexes or cryptic species [19, 20]. Thus, the integration of multiple datasets allows a more comprehensive understanding of the complex evolutionary processes that give rise to unrecognized species [21], such as ecological speciation [22] and incomplete lineage sorting [5]. This approach moves taxonomy beyond species descriptions and provides a more nuanced understanding of phylogenetic relationships within and between taxa.

The genus *Coridius* belonging to the family Dinidoridae is distributed throughout the Afrotropical (22 species), Australasian (1 species), Indomalayan (16 species), and Palearctic (2 species) realms, with 9 species known from India [23, 24]. These are phytophagous insects, primarily feeding on plants of the Arecaceae, Cucurbitaceae, Fabaceae, and Moraceae [25]. Although the host plants of many *Coridius* species are known [25], the life cycle and ecology of most species remain underexplored.

**Fig 1:**
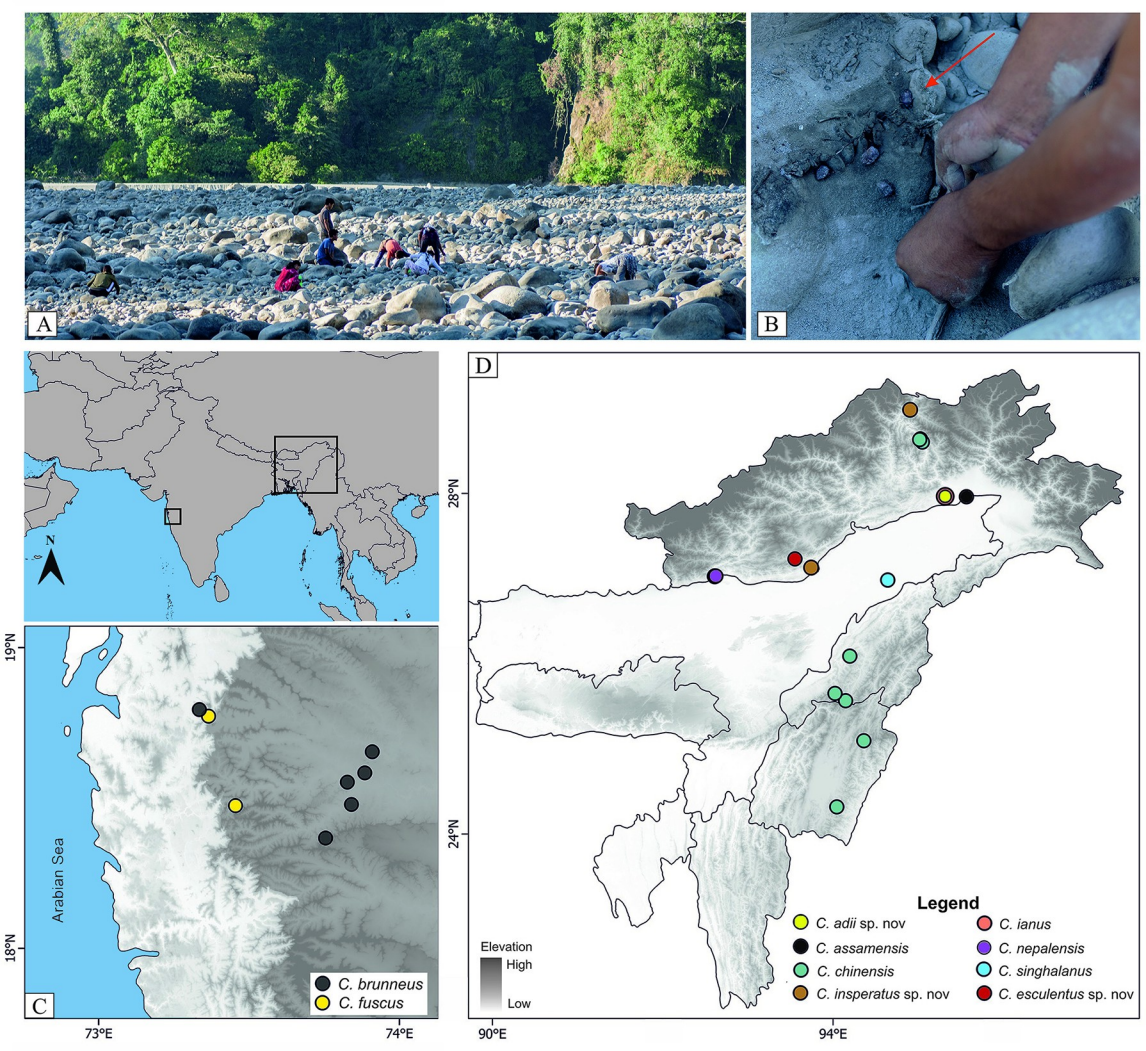
Habitat and collection localities. **(**A) Natural habitat of *Coridius* spp. in Arunachal Pradesh. (B) *Coridius* spp. in a dry riverbed. (C) & (D) Collection localities of *Coridius* spp. across India. The map was created using QGIS Geographic Information System v3.28 by S. S. Boyane and does not include any copyrighted material. The SRTM file used in the map was obtained from the USGS server (public domain).

The genus *Coridius* is economically important, as species of this genus are widely consumed by the native people of northeast India. Members of this genus are also known for their various therapeutic properties [26]. These insects are collected from the dry riverbeds (Fig 1A and 1B), where they diapause during the winter, and are sold in local markets. However, the impact of this widespread consumption of *Coridius* on its population is not very well understood, and a lack of baseline information on the ecology and evolution of this group further hinders the development of any bioprospecting and conservation strategies.

Until 1980, a total of 37 species of *Coridius* were described [27] based on their morphological characteristics, including traits such as coloration and male genitalia. However, several of these species were later synonymized or reassigned to other genera. Due to the intricate taxonomic complexities in *Coridius*, identifying species boundaries based solely on morphological and anatomical characteristics may be inadequate and could lead to further confusion. Moreover, *Coridius* species show a range of host preferences and cryptic morphological differences, making it an ideal study system to apply integrative taxonomic methods and reassess species boundaries.

In this context, to address the taxonomic complexities within the genus *Coridius*, we have set three primary objectives. Firstly, we aim to conduct a comprehensive systematic revision of *Coridius* species found in India, focusing on the highly diverse northeast region with improved sampling. Secondly, we intend to identify key morphological characters to verify previous systematic treatments of the genus. Lastly, we attempt to develop a multilocus phylogeny and employ species delimitation analysis based on molecular data as an additional test to gain confidence in the recognized species groups.

## Material and methods

### Taxon sampling

We collected 150 specimens from October 2018 to January 2021 from 21 different locations across India (Fig 1C and 1D). The collected specimens were preserved in absolute alcohol for morphological and molecular studies.

#### Nomenclatural acts

The electronic edition of this article conforms to the requirements of the amended International Code of Zoological Nomenclature, and hence the new names contained herein are available under that Code from the electronic edition of this article. This published work and the nomenclatural acts it contains have been registered in ZooBank, the online registration system for the ICZN. The ZooBank LSIDs (Life Science Identifiers) can be resolved, and the associated information viewed through any standard web browser by appending the LSID to the prefix “http://zoobank.org/”. The LSID for this publication is: urn:lsid:zoobank.org:pub: 3BCC7D9C-FC18-4A74-B923-4900C8CDAD41.

*Biodiversity board permits*. For this study, all required permits were obtained from the respective biodiversity boards in the respective regions. In Arunachal Pradesh, permit number SFRI/APBB/9/2011/4019, issued by the Arunachal Pradesh Biodiversity Board. In Nagaland, permit number NSBB/31/PABR/2016/652, granted by the Nagaland State Biodiversity Board. In Manipur, the necessary permissions were obtained from the Manipur Biodiversity Board, permit number 3/31/2013-17/APCCF/BIO & NTFP: 67.

### Morphological analysis

The collected specimens were mounted and studied under Zeiss Stemi 305. Photographs and measurements of the specimens were taken using Keyence VHX6000 and processed using Adobe Photoshop 2024. The last two abdominal segments were detached and boiled with 10% KOH for 10 minutes to separate the pygophore. The pygophore was then dissected to separate the parameres. The dissected specimen was briefly rinsed with dilute acetic acid and distilled water, followed by 70% alcohol, and then mounted again.

**Fig 2:**
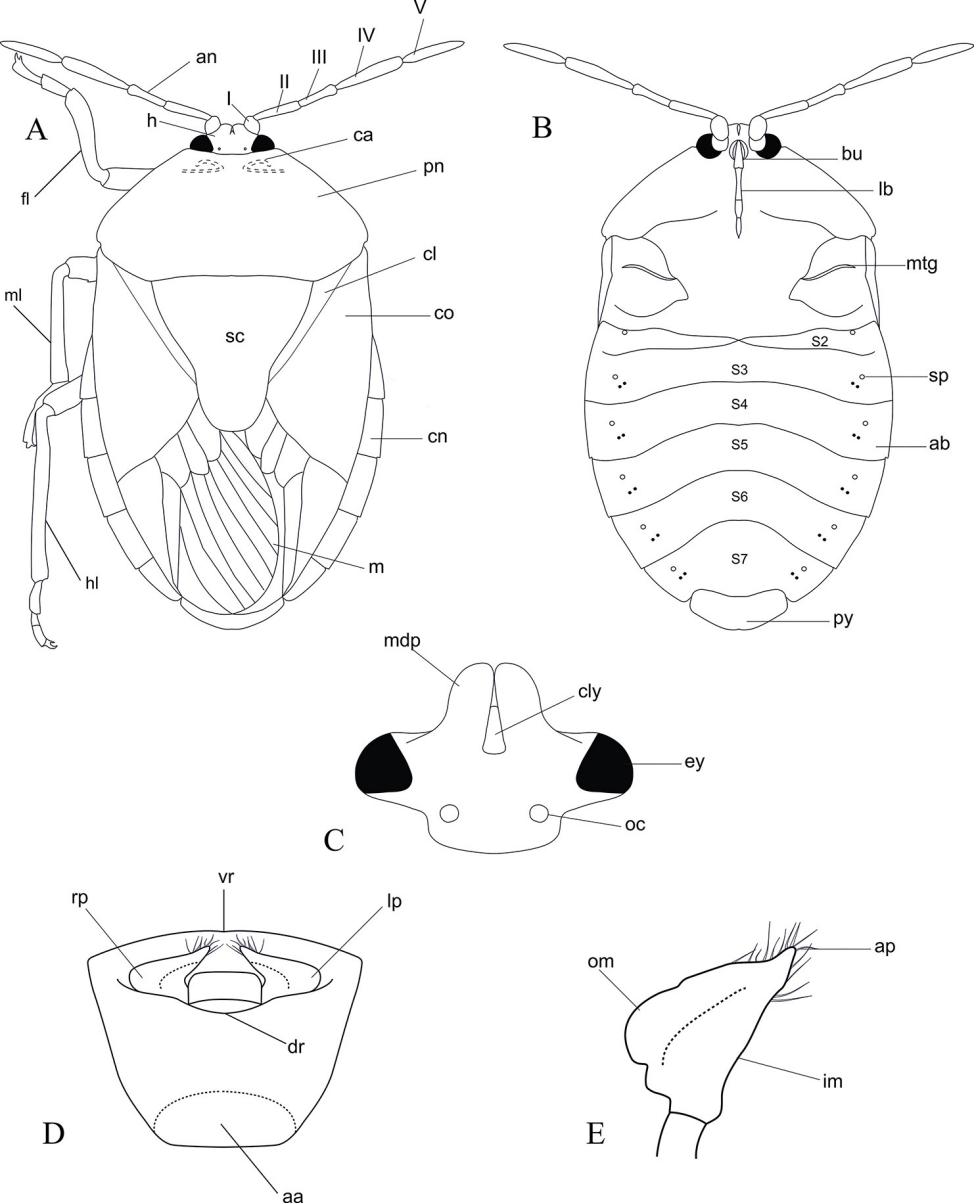
General body morphology of *Coridius* spp. (A) dorsal habitus. (Scale bar = 5 mm) (B) ventral habitus (without legs). (C) head, dorsal view (Scale bar = 1 mm). (D) pygophore, dorsal view (Scale bar = 1 mm). (E) paramere, dorsal view (Scale bar = 1 mm).

Abbreviations used for morphological characters (Fig 2): aa = anterior aperture; ab = abdomen; ap = apical tip; an = antenna; I–V = antennal segments; bu = buccula; ca = calli; co = corium; cl = clavus; cly = clypeus; cn = connexivum; dr = dorsal rim; ey = eye; fl = fore leg; h = head; hl = hind leg; im = inner margin; lb = labium; lp = left paramere; m = membrane; mdp = mandibular plate; ml = mid leg; mtg = meta thoracic scent gland; oc = ocelli; om = outer margin; pn = pronotum; py = pygophore; rp = right paramere; sc = scutellum; sp = spiracle; s_2—_s_7_ = abdominal sternite II–VII. The terminology used herein generally follows that of [28].

Measurements for all species are given in the S1 File.

We measured the head length (HL) and head width to the eye (HW) to calculate the HL/HW ratio; similarly, we measured the length of pronotum (PL) and maximum width of pronotum (PB) to calculate the PL/PB ratio; length of scutellum (SL) and maximum width of scutellum (SB) to calculate the SL/SB ratio; maximum breadth of the body (MB); and total body length (TBL). Linear Discriminant Analysis (LDA) was performed using the morphological characters of 10 species. Two species (*C*. *adii* **sp. nov.,** and *C*. *assamensis*) were dropped from LDA due to insufficient samples. The LDA was performed using R v.4.1.3 packages ‘klaR’ [29], ‘psych’ [30], ‘MASS’ [31], and ‘ggord’ [32].

### DNA extraction and sequencing

Total genomic DNA was extracted from sternal muscles using the QIAGEN DNeasy blood and tissue kit following the manufacturer’s protocol. We PCR amplified and sequenced two mitochondrial loci, Cytochrome C Oxidase Subunit 1 (COI F: 5’ GGTCAACAAATCATAAAGATATTGG 3’, COI R: 5’ AAACTTCAGGGTGACCAAAAAATCA 3’) [33] and 16S rRNA subunit (16s F: 5’ CGCCTGTTTATCAAAAACAT 3’, and 16S R: 5’ CCGGTCTGAACTCAGATCACGT 3’) [34]. The PCR conditions for amplifying the COI consisted of an initial denaturation at 95˚C for 5.00 min, followed by 40 cycles at 94˚C for 30 s, 46˚C for 1.00 min, and 72˚C for 1.45 min, and a final extension at 72˚C for 15.00 min. Similarly, for the 16S initial denaturation, the temperature was set at 95˚C for 5.00 min, followed by 40 cycles of denaturation at 95˚C for 30 sec, an annealing temperature at 53˚C for 1.00 min, and 72˚C for 1.30 min, followed by a final extension at 72˚C for 10 min. We could not sequence any nuclear genes due to the lack of a comprehensive dataset available in GenBank. All PCR products were purified and sequenced in the Barcode Biosciences laboratory (Bengaluru, India). The resulting DNA sequences were deposited in GenBank (see Table 1).

**Table 1:** List of voucher specimens used for molecular-genetic analyses.

Sl. No	Species	Locality and State	Latitude	Longitude	Voucher number	GenBank Accession numbers	
						COI	16s
1	*Coridius brunneus* 1	Charoli, Pune, Maharashtra	18.653°N	73.909°E	AIMB/HE/DIN 000001[E]	OM281737	OM307607
2	*Coridius assamensis* 1	Loklung, Lower Dibang valley, Arunachal Pradesh	27.960°N	95.566°E	AIMB/HE/DIN 000002[E]	OM305088	OM310763
3	*Coridius adii* **sp. nov.**	Rani Village, Pasighat, Arunachal Pradesh	27.9653°N	95.316°E	AIMB/HE/DIN 000003[E]	OM305094	OM321366
4	*Coridius singhalanus* 1	Sivsagar, Assam	26.982°N	94.642°E	AIMB/HE/DIN 000004[E]	OM305092	OM310986
5	*Coridius singhalanus* 2	Sivsagar, Assam	26.982°N	94.642°E	AIMB/HE/DIN 000005[E]	OM305093	OM310987
6	*Coridius nepalensis* 1	Tippi, West Kameng, Arunachal Pradesh	27.026°N	92.606°E	AIMB/HE/DIN 000006[E]	OM283546	OM310994
7	*Coridius nepalensis* 2	Tippi, West Kameng, Arunachal Pradesh	27.029°N	92.623°E	AIMB/HE/DIN 000007[E]	OM283547	OM310995
8	*Coridius nepalensis 3*	Tippi, West Kameng, Arunachal Pradesh	27.026°N	92.606°E	AIMB/HE/DIN 000008[E]	OM283548	OM310996
9	*Coridius insperatus* **sp. nov.** 1	Yingkiomg, Upper Siang, Arunachal Pradesh	28.635°N	95.024°E	AIMB/HE/DIN 000009[E]	ON220867	OM321361
10	*Coridius insperatus* **sp. nov.** 2	Tippi, West Kameng, Arunachal Pradesh	27.026°N	92.610°E	AIMB/HE/DIN 000011[E]	ON220869	OM321363
11	*Coridius insperatus* **sp. nov.** 3	Nirjuli, Papum Pare, Arunachal Pradesh	27.128°N	93.742°E	AIMB/HE/DIN 000012[E]	ON220870	OM321364
12	*Coridius insperatus* **sp. nov.** 4	Nirjuli, Papum Pare, Arunachal Pradesh	27.128°N	93.742°E	AIMB/HE/DIN 000013[E]	ON231399	OM321365
13	*Coridius esculentus* **sp. nov.**	Nimte, Papum Pare, Arunachal Pradesh	27.231°N	93.552°E	AIMB/HE/DIN 000016[E]	ON209539	OM311263
14	*Coridius chinensis* 1	Ukhrul, Manipur	25.095°N	94.361°E	AIMB/HE/DIN 000014[E]	ON209538	OM311261
15	*Coridius chinensis* 2	Ukhrul, Manipur	25.095°N	94.361°E	AIMB/HE/DIN 000015[E]	ON222735	OM311262
16	*Coridius chinensis* 3	Viswema, Kohima, Nagaland	25.565°N	94.147°E	AIMB/HE/DIN 000017[E]	ON209540	OM311264
17	*Coridius chinensis* 4	Viswema, Kohima, Nagaland	25.565°N	94.147°E	AIMB/HE/DIN 000018[E]	ON222736	OM311265
18	*Coridius chinensis* 5	Khonoma, Kohima, Nagaland	25.651°N	94.022°E	AIMB/HE/DIN 000019[E]	OM319823	OM311266
19	*Coridius chinensis* 6	Kamro, Upper siang, Arunachal Pradesh	28.602°N	95.048°E	AIMB/HE/DIN 000020[E]	OM319824	OM311267
20	*Coridius chinensis* 7	Chandel, Manipur	24.318°N	94.044°E	AIMB/HE/DIN 000021[E]	OM319825	OM311268
21	*Coridius chinensis* 8	Elumyo, Wokha, Nagaland	26.089°N	94.196°E	AIMB/HE/DIN 000022[E]	OM319826	OM311269

### Sequence alignments

Sequences were obtained from 22 specimens consisting of eight species (Table 1). Additionally, we retrieved 19 sequences of *Coridius* and 20 sequences belonging to Pentatomoidea and Lygaeoidea and treated them as outgroups from the GenBank. The sequenced chromatograms were manually inspected for quality and aligned using the software program Geneious Version 2023.1.1. The gaps in sequences were treated as “missing”.

### Phylogenetic analysis

The phylogenetic analysis was conducted based on a total of 61 sequences from 11 species of *Coridius*. The final combined alignment consists of 1002 nucleotides, of which 248 sites were parsimony informative. The best-fit nucleotide substitution model for Bayesian inference was selected for both genes based on the Bayesian information criterion (BIC) in PartitionFinder 2.0 [35] (Table 2). The HKY+I+G substitution model was assigned to the first codon position, and the TRN+G model was assigned to the second and third codon positions for COI. The HKY+I+G substitution model was assigned for the first, second, and third codon positions in 16s. We ran maximum-likelihood (ML) analyses in IQ-Tree Web Serve [36] with 1000 bootstrap replicates under the GTRGAMMA substitution model in the combined dataset. A Bayesian inference (BI) was performed in the software program MrBayes 3.2.1 [37]. Four independent chains of Markov chain Monte Carlo (MCMC) were performed for 50 million generations with a sampling frequency of 500. The convergence of MCMC chains was assessed in Tracer [38]. The first 25% of the runs were discarded, and the remaining trees were used to build a consensus tree and estimate Bayesian posterior probabilities (PP). We used FigTree 1.4.4 [39] to visualize and edit the phylogenetic trees.

**Table 2:** Partition schemes and substitution models used for the Maximum likelihood (ML), Bayesian Inference (BI).

Sl. no	Gene	RaxML	Mr Bayes	Length (bp)
1	COX1 codon 1	GTRGAMMA	HKY+I+G	187
2	COX1 codon 2	GTRGAMMA	TRN+G	186
3	COX1 codon 3	GTRGAMMA	TRN+G	186
4	16S codon 1	GTRGAMMA	HKY+I+G	148
5	16S codon 2	GTRGAMMA	HKY+I+G	148
6	16S codon 3	GTRGAMMA	HKY+I+G	147
	Combined			1002

#### Species delimitation

Molecular species delimitation analyses were conducted using the Bayesian Poisson Tree Processes (bPTP) method through the online web server available at http://species.h-its.org/. The analyses consisted of 100,000 Markov Chain Monte Carlo (MCMC) generations with a thinning parameter set to 100 and a post-burn-in value of 0.1. Additionally, a single-rate PTP analysis was performed using the ML tree generated from IQTREE (https://mptp.h-its.org/#/tree), with the default p-value settings [40]. To further support our species delimitation method, we employed the Assemble Species by Automatic Partitioning (ASAP) analysis, utilizing the genetic distance matrix method [41]. This analysis was conducted through a web-based interface accessible at https://bioinfo.mnhn.fr/abi/public/asap/, after setting the default parameters. For this analysis, we selected the Kimura (K80) (TS/TV) 2.0 model. The best two ASAP scores resulting from this analysis have been referred to as ASAP 1 and ASAP 2.

## Results

### Linear Discriminant Analysis (LDA)

**Fig 3:**
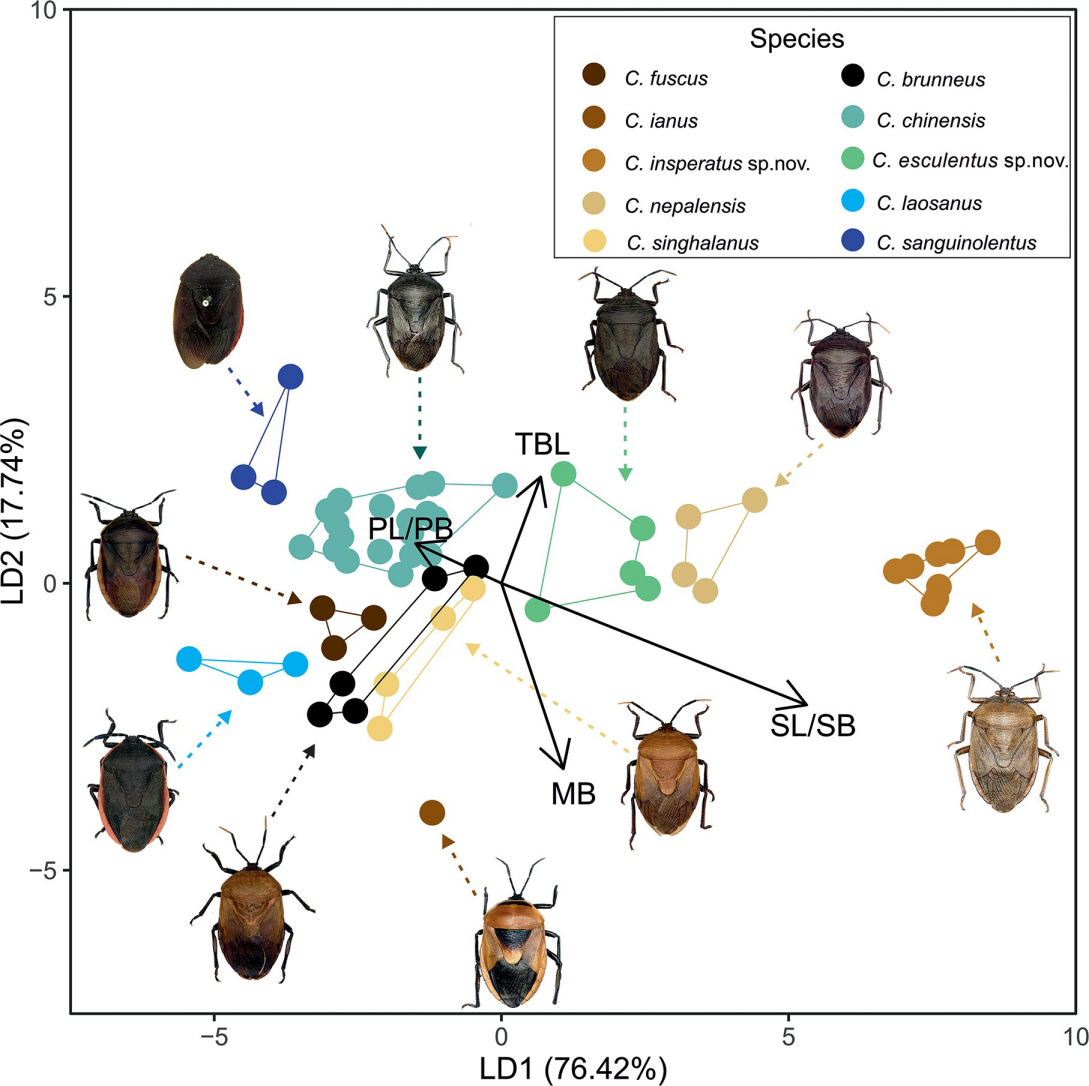
Linear Discriminant Analysis of six selected morphological characters based on the 10 species. The percentage separation achieved by the first two linear discriminant functions is 76.42% for axis 1 and 17.74% for axis 2, respectively. Because of the limited sample, *C*. *adii* **sp. nov.,** and *C*. *assamensis* (Distant, 1902) were excluded from LDA.

The first two linear discriminant axes for the LDA are presented in Fig 3. These axes cumulatively explain 94.16% of the total variance (76.42% and 17.74%, respectively). Based on prior knowledge, we assigned ten groups, out of which eight were distinctly clustered. These clusters include the species *C*. *chinensis* (Dallas, 1851), *C*. *esculentus* **sp. nov.,** *C*. *insperatus* **sp. nov.,** *C*. *fuscus* (Westwood, 1837), *C*. *ianus* (Fabricius, 1775), *C*. *laosanus* (Distant, 1921), *C*. *nepalensis* (Westwood, 1837), and *C*. *sanguinolentus* (Westwood, 1837). On the other hand, *C*. *brunneus* (Thunberg, 1783) and *C*. *singhalanus* (Distant, 1900) do not form distinct clusters, as they overlap with each other (Fig 3). Four characters that were morphologically significant in species differentiation were recognized by the LDA. These include the total body length (TBL), the maximum body breadth (MB), and the pronotum and scutellum’s length and breadth ratios (PL/PB and SL/SB, respectively). The scatter diagram, histogram of the distribution of a single variable, and correlation values for each variable are provided in S1 and S2 Figs.

### Phylogenetic inference

**Fig 4:**
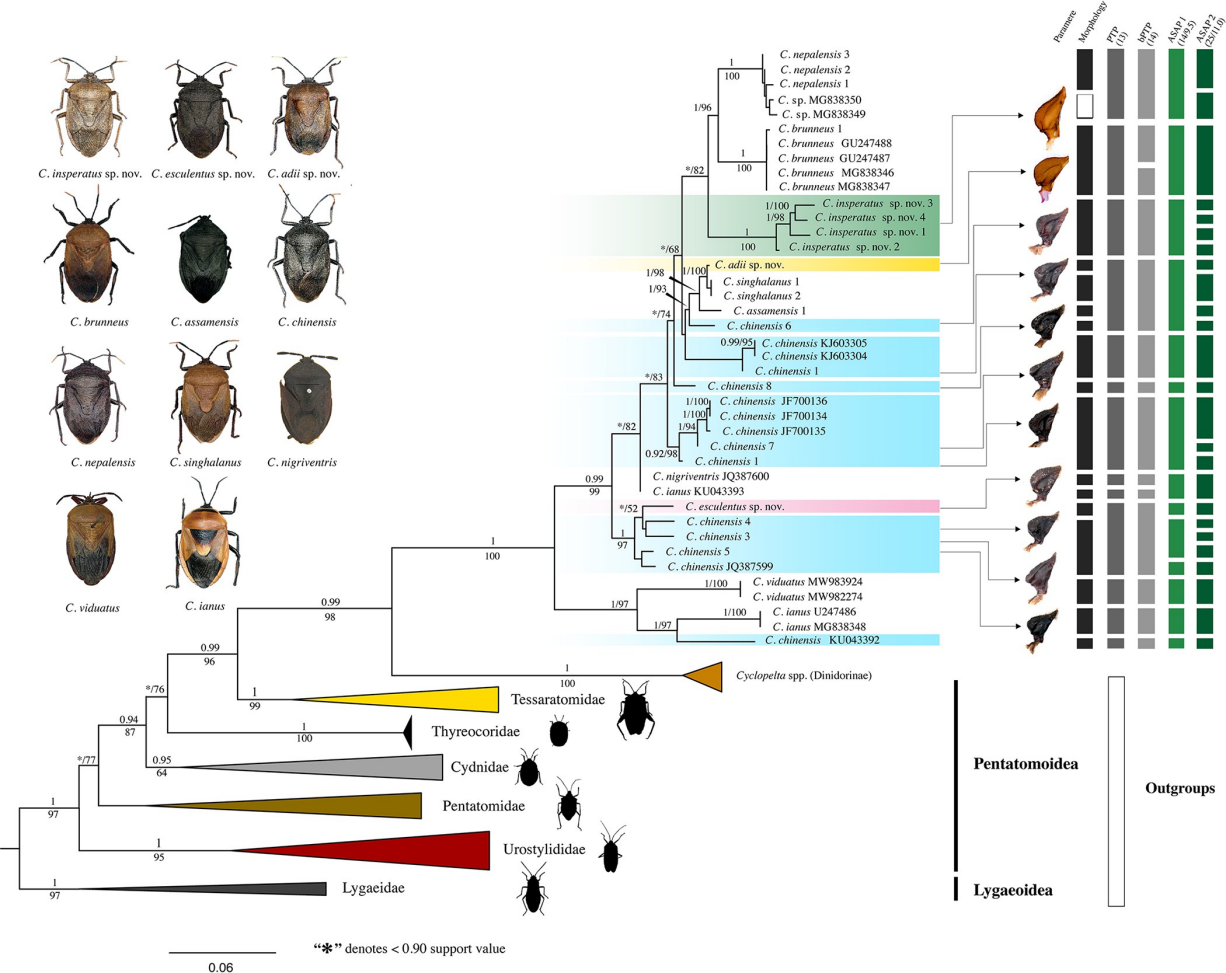
Bayesian phylogenetic tree based on mtDNA markers. Support values above and below nodes: Bayesian posterior probabilities (top) with <0.90 denoted as (*), and maximum likelihood bootstrap values (bottom). On the right side of the figure, morpho-species assignments and the results of species delimitation tests are displayed: PTP (13 taxa grouping), bPTP (14 taxa grouping), ASAP 1 (14 taxa grouping, with an ASAP score of 9.5), and ASAP 2 (25 taxa grouping, with an ASAP score of 11.0). A white gap in the morphology column indicates the unavailability of voucher specimens. Parameres for *C*. *adii* **sp. nov.,** *C*. *chinensis* complex, *C*. *esculentus* **sp. nov.,** and *C*. *insperatus* **sp. nov.** are shown (for more information on parameres, refer to Figs 12 and 17).

Maximum Likelihood (ML) and Bayesian Inference (BI) tree analyses of two concatenated genes (COI and 16S) showed similar topologies with high support (maximum likelihood bootstrap support, MLBS) values and posterior probabilities (PP) (Fig 4). Lygaeidae was used as the root for the tree. The family-level relationship showed Urostylididae (PP: 1, MLBS: 95) as the sister taxa to all other Pentatomoidea in both analyses. Also, we found clear evidence of Tessaratomidae (PP: 1, MLBS: 99) being a sister taxon to Dinidoridae (PP: 0.99, MLBS: 96). Furthermore, the monophyly of Thyreocoridae (PP: 0.62/76), Cydnidae (PP: 0.94, MLBS: 87), and Pentatomidae (PP: 0.62, MLBS: 77) were consistent in both analyses. Finally, Pentatomidae (PP: 0.62, MLBS: 77) were recovered as the sister taxon to the group comprising Cydnidae, Thyreocoridae, Tessaratomidae, and Dinidoridae.

The species-level relationship showed the monophyly of *C*. *insperatus* **sp. nov.** (PP: 1, MLBS: 100) and *C*. *nepalensis* (PP: 1, MLBS: 100) received strong support, indicating their distinct evolutionary lineages. Similarly, *C*. *singhalanus* (PP: 1, MLBS: 100) and *C*. *brunneus* (PP: 1, MLBS: 100) were supported as monophyletic groups. Moreover, *C*. *adii* **sp. nov.** (PP: 0.99, MLBS: 99) was found to be closely related as a sister group to *C*. *singhalanus* (PP: 1, MLBS: 98). In our analysis, we found that *C*. *nigriventris* is in a polytomy with one specimen of *C*. *ianus*. The most widespread species within this genus, *Coridius chinensis*, is paraphyletic, with at least six clades. Interestingly, *C*. *esculentus* **sp. nov.** was found to be grouped within a clade alongside *C*. *chinensis* but formed a distinct branch (PP: 0.82, MLBS: 52). This outcome was in accordance with our expectations, as *C*. *chinensis* and *C*. *esculentus* **sp. nov.** have similar coloration but differ significantly in anatomy (see the discussion section and Fig 4). Furthermore, our investigation revealed that the African species *C*. *viduatus* and *C*. *ianus* form a sister group (PP: 1, MLBS: 100), aligning with our expectations due to their striking morphological similarity (Fig 4).

#### Species delimitation and new lineages

The estimated molecular operational taxonomic units (MOTUs) were not consistent among the methods: 13 for PTP, bPTP for 14, ASAP 1 for 14, and ASAP 2 for 25 (excluding out-groups) (Fig 4). The species status of *C*. *insperatus* **sp. nov.** was supported by the analysis. *Coridius nepalensis* and all undetermined species were treated as a single species in the analysis. In the analysis, PTP, bPTP, and ASAP 1 suggested grouping four species, namely *C*. *singhalanus*, *C*. *adii* **sp. nov.**, *C*. *assamensis*, and *C*. *chinensis*, as a single species, whereas ASAP 2 proposed each of these species should be considered distinct species. Similarly, *C*. *esculentus* **sp. nov.** is found nested with four other individuals of *C*. *chinensis*, forming a well-supported clade in the phylogenetic analysis (PP: 0.99, MLBS: 95). Both ASAP 1 and ASAP 2 have recognized them as distinct species. *Coridius nigriventris* (JQ387600) clustered with *C*. *ianus* (KU043393) in ML and BI topologies, while the PTP and bPTP analyses suggest these to be two separate species.

Interestingly, our analysis showed that *C*. *chinensis* belongs to a taxonomic species complex forming six different lineages. The first lineage was composed of a single specimen found in Arunachal Pradesh. The second lineage included two specimens from Guizhou Province, China (KJ603304 and KJ603305) and one from Manipur, India. The third lineage consisted of a single specimen found in Nagaland. The fourth lineage was formed from two specimens from Manipur and three from China (JF700134, JF700135 and JF700136) with strong node support (PP: 0.99, MLBS: 98). The fifth lineage included three specimens from Nagaland (*C*. *chinensis* 4; *C*. *chinensis* 3; and *C*. *chinensis* 5) and *C*. *chinensis* (JQ387599) from China. The sixth lineage consisted of a single specimen from Kerala, India, *C*. *chinensis* (KU043392).

### Taxonomic treatment

       Family Dinidoridae Stål, 1870

         Subfamily Dinidorinae

           Tribe Dinidorini

        Genus *Coridius* Illiger, 1807

*Coridius* llliger, 1807: 361; Schumacher, 1924: 335; Bergroth, 1927: 7; Yang, 1940: 6–10; Hoffmann, 1948: 21, 22; Stichel, 1962: 725; Nuamah, 1982: 24, 25; Durai, 1987: 185–190; Rolston *et al*., 1996: 31–33, 97; Lis, 1996: 51–54, Figs 1–8; Ahmad *et al*., 1997: 305–320, Figs 1–4; Kocorek, 2003: 51–55, Figs 1–5. Type species: *Cimex ianus* Fabricius, 1775 by monotypy.

*Aspongopus* Laporte, 1832: 58; Burmeister, 1835: 347, 351; Spinola, 1837: 304, 305; Amyot & Serville, 1843: XXIX, 173; Herrich-Schäffer, 1844: 77; Agassiz, 1846: 3; Spinola, 1850: 33; Dallas, 1851: 318, 348; Herrich-Schäffer, 1851: 282; Fieber, 1860: 79; 1861: 330; Stål, 1865: 81, 212; Mulsant & Rey, 1866: 234; 1867: 157; Stål, 1867: 522; Vollenhoven, 1868: 38; Walker, 1868: 480; Stål, 1872: 40; Puton, 1886: 15; Atkinson, 1889: 87; Lethierry & Severin, 1893: 236; Distant, 1902: 279, 281; Oshanin, 1906: 161; Bergroth, 1908: 188; Kirkaldy, 1909: 255; Schouteden, 1909: 71; Oshanin, 1912: 19; Jeannel, 1913: 100; Kirkaldy, 1913: 84; Schouteden, 1913: 6, 7; Hesse, 1925: 41; Tang, 1935: 335; Cachan, 1952: 294; Villiers, 1952: 86; Priesner & Alfieri, 1953: 12; Cobben, 1968: 112, Figs 114A, 114B; Hsiao et al., 1977: 69, 70. Type species: *Cimex janus* Fabricius, 1775, by monotypy. Synonymized by Schumacher, 1924: 335.

*Spongopodium* Spinola, 1837: 305; Agassiz, 1846: 18; Spinola, 1850: 33. Type species: *Cimex obscurus* Fabricius, 1794, by monotypy. Synonymized by Stål, 1865: 212.

*Amacosia* Spinola, 1850: 78. Type species *Amacosia delegorguei* Spinola, 1850 by monotypy. Synonymized by Walker, 1864: 480.

*Aspongobus* [sic] Dohrn, 1859: 21.

*Peltagopus* Signoret, 1861: 936. Type species: *Peltagopus flavomarginatus* Signoret 1861 by monotypy. Synonymized by Stål, 1865: 212.

*Aspongopus* (*Aspongopus*) Stål, 1870: 81; Schouteden, 1913: 7.

Synonymy used here and henceforth is based on Lis [24], Durai [27], and Rolston et al. [42].

#### Diagnosis

Head small, triangular, broader than long, mandibular plates longer than clypeus, rounded, and meeting or not in front of clypeus. Ocelli prominent, placed closer to eyes than to each other. Eyes globular, pedunculate. Labium four segmented, reaching about middle of meso-sternum, first and second segment mostly sub-equal, third and fourth smaller and sub-equal. Bucullae prominent, raised above the level of labium. Antennae four or five segmented, first segment surpassing apex of head, second and third subequal or sometimes second longer or shorter than third, fourth generally long with setae on both sides, fifth cylindrical, and finely setose. In some species second, third and fourth antennal segments grooved and flattened.

Pronotum declivous, trapezoidal. Lateral margins straight or rounded, sometimes slightly raised or carinate; dorsally rugulose punctate. Calli rugulose and smooth. Anterior margin concave behind head. Anterior angles slightly broader than width at eye. Humeral angles rounded, slightly truncate. Posterior margin moderately straight over scutellum.

Scutellum broader than long, longer than clavus, lateral margin distinctly sinuate, and its apical region broadly rounded.

Hemelytra longer than abdomen. Clavus narrow, short, corium broad. Membrane long and broad with numerous longitudinal veins.

Pro-sternum deeply sulcate mesially, its lateral area distinctly raised or flat, posterior margin slightly sinuate in lateral region. Pro-coxal area distinctly raised, pro-coxae very close together. Meso-sternum smooth on disk with median groove and longitudinal rugae. Meta-sternum very narrow between coxae. Metathoracic scent gland situated close to metacoxae, more ventral in position with narrow peritreme and well-developed evaporatoria.

Coxae close together in a linear series. Femora slightly curved or straight with spines underneath in distal half, tibiae distinctly sulcate and with spines distally. First segment of tarsus more setose, second and third with very sparse, long setae. Claws divergent, pulvilli distinct, well developed. Hind tibia in females slightly dilated and with oval tympanal organ.

Abdomen broad, connexivum moderately or well exposed. Segmental sutures sinuate, seventh sternum longest. Spiracles black, situated closer to anterior margin than lateral margin of segment. Spiracle of first visible abdominal segment partially or totally covered by metasepimeron. Trichobothria distributed in pairs posterior to pseudosutures and spiracle.

Pygophore visible from dorsal as well as from ventral sides, basally convex, flat in distal half, ventral surface rugulose; ventral rim rounded or notched medially or sometimes raised or concave or straight; dorsal rim moderately concave or straight. Parameres short or slender, apical tip rounded or blunt or pointed.

**Fig 5:**
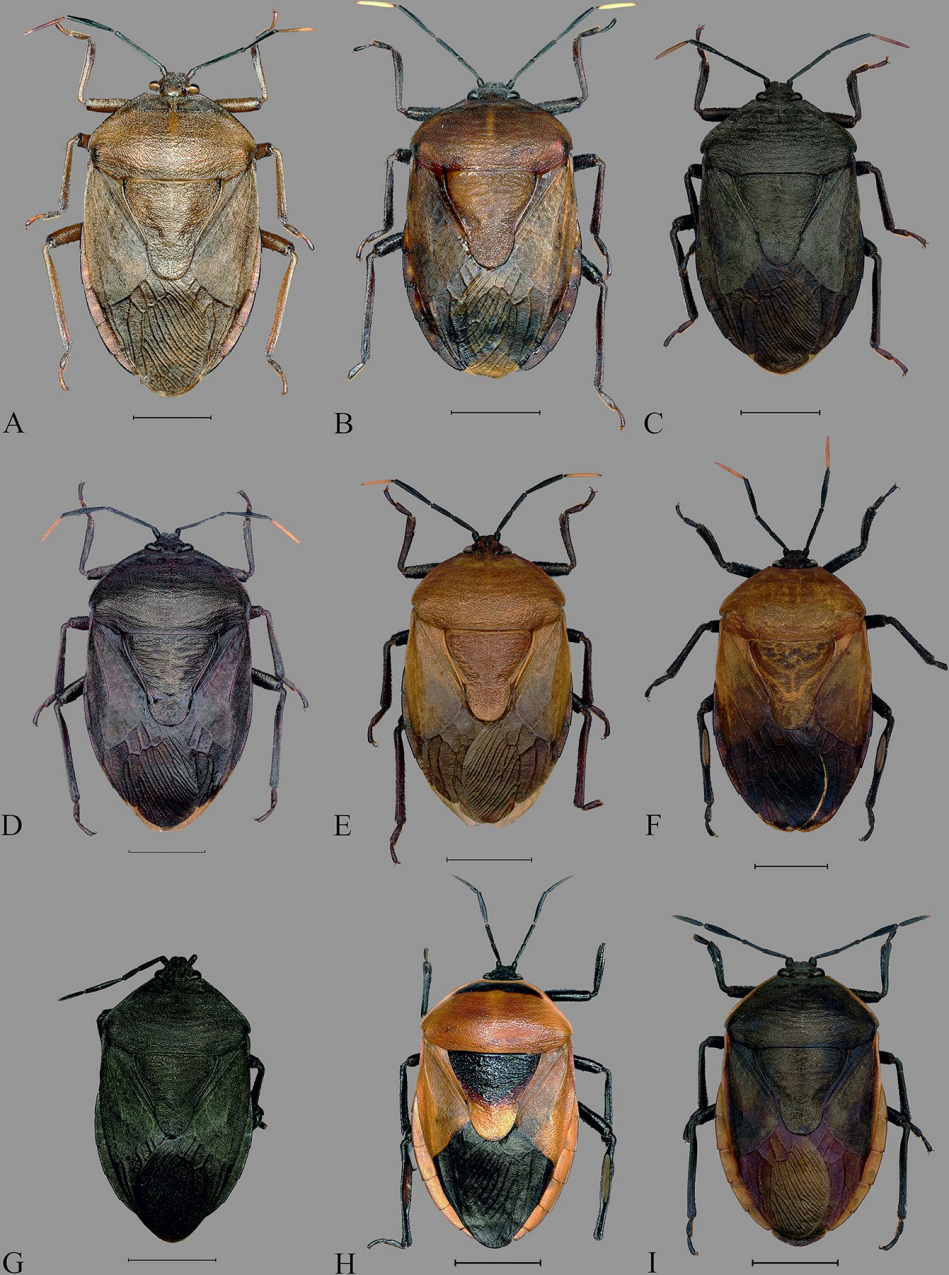
**Dorsal habitus of *Coridius* spp. (**A) *C*. *insperatus* **sp. nov.**, (B) *C*. *adii* **sp. nov.**, (C) *C*. *esculentus* **sp. nov.**, (D) *C*. *nepalensis*. (E) *C*. *singhalanus*. (F) *C*. *brunneus*. (G) *C*. *assamensis* (antennae mutilated). (H) *C*. *ianus*. (I) *C*. *fuscus*. (Scale bar = 5 mm).

**Fig 6:**
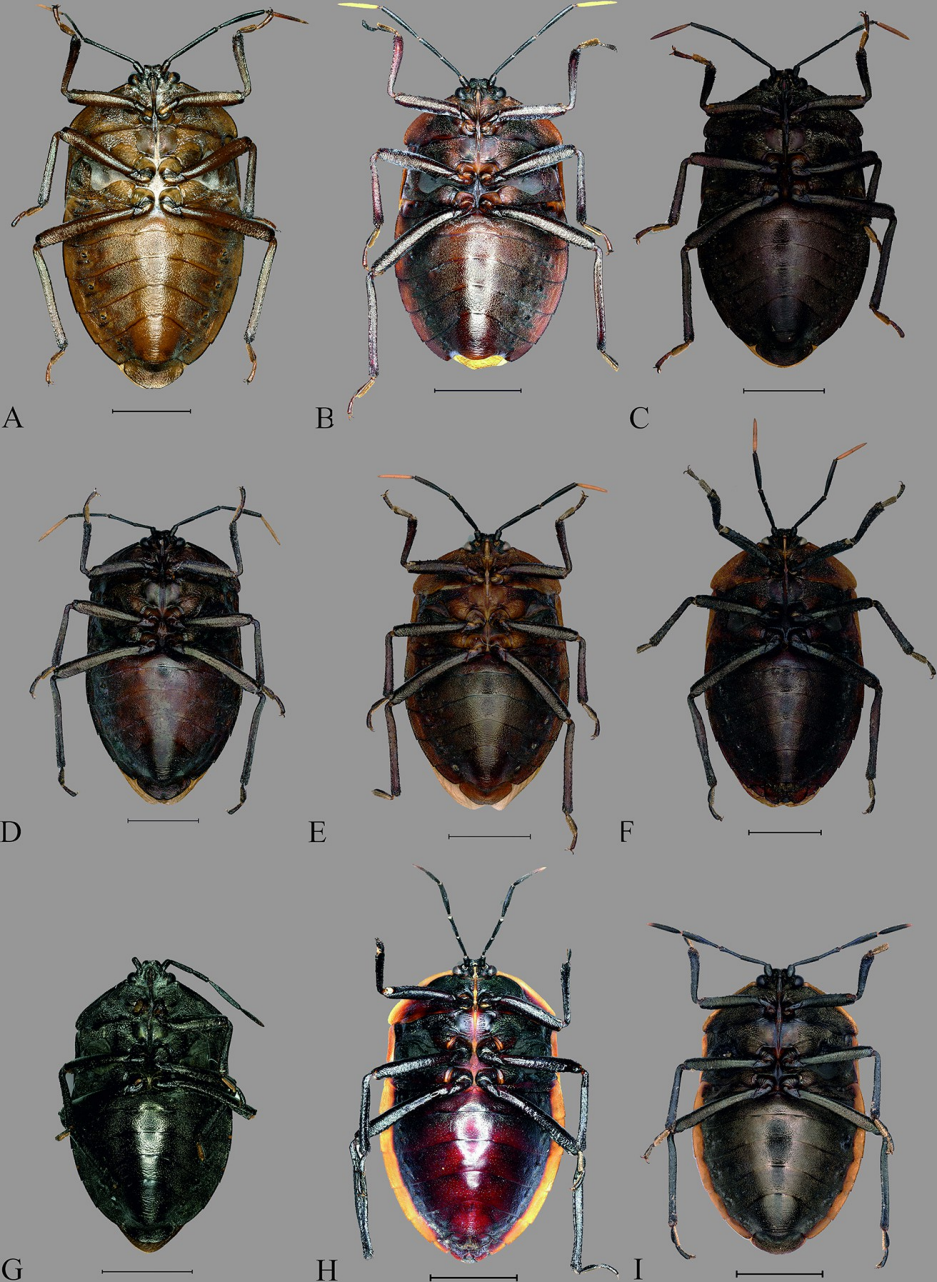
Ventral habitus of *Coridius* spp. (A) *C*. *insperatus* **sp. nov.**, (B) *C*. *adii* **sp. nov.**, (C) *C*. *esculentus* **sp. nov.**, (D) *C*. *nepalensis*. (E) *C*. *singhalanus*. (F) *C*. *brunneus*. (G) *C*. *assamensis* (antennae mutilated). (H) *C*. *ianus*. (I) *C*. *fuscus*. (Scale bar = 5 mm).

***Coridius insperatus* Boyane, Ghate & Priyadarsanan, sp. nov.** urn:lsid:zoobank.org:act:69C0D1FA-D2E0-4CD6-A1B3 9CAEE189EA15 (Figs 5A, 6A, 9A, 10A, 11A and 12A).

*Etymology*. The species name is the Latin adjective *insperatus* (meaning ‘unexpected’ in Latin) because it is an unexpected member of the genus *Coridius* with four segmented antennae.

*Material examined*: *Holotype male*. INDIA: Arunachal Pradesh, Nirjuli, leg. Anil Talang, 1.i.2019, 27.128°N, 93.742°E, 139 m.

*Paratypes*. Same collecting data as holotype: 1 F.—INDIA: Arunachal Pradesh, Tippi, leg. Thejavikho Chase, 30.xi.2020, 27.026°N, 92.610°E, 179 m: 1 F.—INDIA: Arunachal Pradesh, Tutting, leg. Gumnya Ete, 13.xii.2019, 28.982°N, 94.905°E, 485 m: 1 F.—INDIA Arunachal Pradesh, Yingkiong, leg. Gumnya Ete, 12.xii.2019, 28.635°N, 95.024°E, 275 m: 1M.

*Diagnosis*. Overall colour yellowish brown. Pronotum ventrally pale yellowish brown, lateral margins black. Legs yellowish brown, femora slightly darker. Antennae with first segment basally ochraceous, distally dark brown, second and third black, fourth ochraceous except a minute region at base and apex which are black. Pronotum with a levigate pale line from posterior region of calli to posterior pronotal margin (Fig 5A). Spiracles black, encircled by a pale ring.

*Description*. Overall structure, Body finely punctate; pronotum with a few transverse rugae in posterior two thirds; region around calli smooth, shining, with sparse punctures. Scutellum rugulose punctate in basal two third, apical region granulated. Prosternum with a distinct elevated smooth region on either side of median groove, laterally rugulose and coarsely punctate. Meso- and meta-sterna smooth medially, rugulose and coarsely punctate in pleural regions. Body nearly smooth medially on abdominal sternites, very finely punctate laterally. Legs laterally compressed; tibiae distinctly sulcate and spinose dorsally and ventrally, more compressed than femora; femora with ventral spines; first segment of tarsus densely setose, second and third with very sparse, long setae; claws widely separated, dark brown to black in distal half; pulvilli distinct, well developed (Figs 5A and 6A).

Head transverse, two times wider than longer at eyes, mandibular plates much longer than clypeus, parallel sided, rounded anteriorly and reaching in front of clypeus (Fig 9A). Dorsal surface of mandibular plates with irregular folds and scattered punctures. Vertex coarsely punctate medially, with longitudinal rugae and grooves near eyes. Eyes large, globular and pedunculate. Ocelli well developed, slightly elevated. Interocellar distance 1.51 times distance between eyes and ocelli. Antennae four segmented, first segment cylindrical with few setae, surpassing apex of head, second and third distinctly laterally compressed, second segment with many setae and longitudinal grooves in basal one fourth with fine spine like setae on the sides, third segment finely rugulose, with spine-like setae on both sides, slightly dilated in basal half while fourth segment rounded, gradually tapering, finely punctate and covered with numerous small and sparse, long setae.

Labium four segmented, reaching middle of meso-sternum; first and second segments sub-equal, third (0.81 mm) and fourth (0.64 mm) smaller. Bucculae prominent, raised above the level of labium.

Pronotum rugulose punctate, calli less punctate, somewhat shining. Anterior margin of pronotum concave behind head, slightly truncate medially behind eyes. Width at anterior angles of pronotum only slightly wider than width at eyes. Lateral margins straight, slightly raised. Humeral angles rounded, posterior margin straight. Prosternum deeply sulcate in middle, its sides distinctly raised. Posterior margin of pro-sternum slightly sinuate in lateral parts. Pro-coxal area distinctly raised, pleural area coarsely punctate and rugulose, closer to coxae. Mesosternum smooth medially with shallow groove in anterior three fourth and slightly deeper groove in distal one fourth, especially near meso coxae. Pleural area coarsely punctate and rugulose. Metasternum very narrow between coxae, metasternum not covering spiracle of first visible abdominal segment posteriorly, leaving it partly exposed.

Scutellum triangular, median region elevated, lateral margins distinctly sinuate in the middle, apical region broadly rounded. Apical area finely punctate and rugulose, with coarse punctures. Median levigate line in basal half continuing pronotal levigate line.

Hemelytra. Clavus narrow, short, corium very broad, very finely and closely punctate and rugulose, punctures at margins slightly coarse. Membrane long and broad with numerous longitudinal veins and three basal cells. Hemelytra longer than abdomen. Extreme basal region of corium upturned forming shallow groove, its margin black, similar to that of pronotum, remaining margin of corium flat.

Abdomen broad, with sinuate intersegmental sutures sinuate, seventh sternum longest. Spiracles situated closer to anterior margin than to lateral margin of segment. Trichobothria distinct, a pair situated posteriorly to pseudosuture, which is posterior to spiracle.

Male genitalia. Pygophore 0.91 times wider than long, somewhat rectangular, and sclerotized. Basally convex, flat in distal half, ventral surface rugulose and ventral margin with fine black denticulation, with sparse setae. Ventral rim with rugae and numerous long ochraceous setae (Fig 10A), ventrally convex and smooth (Fig 11A). Parameres sclerotized, short, medially rounded and apically narrowing, apical tip with rugae and setae (Fig 12A).

*Geographical distribution*. Presently known only from Arunachal Pradesh, India.

*Remarks*. *Coridius insperatus* **sp. nov.,** is a very distinct species compared to other congeneric species. It can be easily distinguished by its four segmented antennae and cupreous dorsal colouration, while all other species in the genus are dark brown, yellow to black dorsally, and have five segmented antennae. In the family Dinidoridae, the genus *Amberiana* Distant, 1911, has one species with four segmented antennae (*A*. *major* Schouteden, 1912), while other species (*A*. *montana* Distant, 1911) have five segmented antennae [23, 24].

This species is edible and consumed by the Nyishi and Adi tribes of Arunachal Pradesh. In addition, we found that this species is sold in the Nirjuli local market (Dist. Itanagar, Arunachal Pradesh) during the winter season (October–January).

***Coridius adii* Boyane, Ghate & Priyadarsanan, sp. nov.** urn:lsid:zoobank.org:act:2A940B0F-7D83-4975-83EE-04718495F7C9 (Figs 5B, 6B, 9B, 10B, 11B and 12B).

*Etymology*. This species is dedicated to the Adi tribe, one of the populous groups of Arunachal Pradesh, inhabiting mainly along the Siang valley, who consume this species for food.

*Material examined*: *Holotype male*. INDIA: Arunachal Pradesh, Rani Village, Pasighat, leg. Swapnil Boyane, 1.x.2018, 27.9653°N, 95.316°E, 139 m. Female unknown.

*Diagnosis*. Overall pale brown to dark brown with irregular patches of yellow on pronotum, and corium. Lateral margins of pronotum black, pronotum with a levigate pale line from posterior region of calli to mid-point of its length. First four segments of antennae black, minute regions at base and apex are ochraceous, fifth ochraceous except a minute region at base and apex which are brown (Figs 5B and 6B). Connexival segments with yellow spot in between segmentation; ventrally, dark brown. Labium pale brown. Pleural area and area near spiracles dark brown or blackish brown. All legs yellowish brown, femora slightly darker, claws dark brown to black in distal half.

*Description*. Overall structure. Body finely punctate, region around calli smooth, shining; scutellum punctate rugulose, its apical region somewhat rounded. Prosternum with smooth flat region on either side of the median groove, laterally rugulose and coarsely punctate. Meso and metasternum smooth.

Head transverse, 1.92 times wider than long, mandibular plates longer than clypeus, rounded anteriorly and meeting in front of clypeus (Fig 9B), dorsally with irregular folds and scattered punctures and very sparse golden setae. Eyes globular and pedunculate. Ocelli prominent, interocellar distance 2.16 times distance between eyes and ocelli. Antennae five segmented, first segment (0.87 mm) cylindrical with sparse setae surpassing apex of head, second (1.59 mm) and third (1.64 mm) subequal, compressed; second, third and fourth segments with longitudinal grooves and fine spine-like setae on the sides, fourth segment slightly dilated in basal half, fifth finely covered with numerous small and sparse long setae, pointed at the tip.

Labium four segmented, reaching anterior third of mesosternum. First (0.94 mm) and second (1.04 mm) segments subequal, third (0.80 mm) and fourth (0.72 mm) smaller, sub-equal. Bucculae prominent, raised above the level of labium.

Pronotum declivous, rugulose, punctate, somewhat shining. Anterior margin of pronotum concave behind head. Lateral margins almost straight, slightly raised. Humeral angles rounded, posterior margin of pronotum straight. Prosternum sulcate in the middle, its lateral area flat. Meso and metasternum smooth on disk with a shallow, rugulose median groove.

Scutellum 0.95 times longer than broad, apex broadly rounded, finely punctate, and longitudinally rugulose.

Hemelytra shorter than abdomen, clavus narrow, short, corium broad, finely, and closely punctate, rugulose. Membrane long and broad with numerous longitudinal veins. Legs laterally compressed; femora with spines ventrally, tibiae distinctly sulcate and spined ventrally as well as dorsally; first segment of tarsus densely setose, second and third with very sparse long setae; claws widely separated, pulvilli distinct, well developed.

Abdomen broad, seventh sternum longest. Spiracles black, closer to anterior margin than lateral margin of respective segment. Trichobothria distinct with a pair situated behind the pseudosuture, posterior to spiracle.

Male genitalia. Pygophore longer than broad, sclerotized, basally convex, flat in distal half. Ventral rim slightly notched, irregular and without setae (Fig 10B), ventrally convex, smooth (Fig 11B). Parameres broad, medially rounded, irregular on outer margin and apically narrowing with numerous long setae (Fig 12B).

*Geographical distribution*. India. This species is known only from its type locality (Pasighat: Arunachal Pradesh).

***Coridius esculentus* Boyane, Ghate & Priyadarsanan sp. nov.** urn:lsid:zoobank.org:act:47A61F00-13F6-42C2-A9AF-A1C1BB853657 (Figs 5C, 6C, 9C, 10C, 11C and 12C).

*Etymology*. The species name ‘*esculentus*’ is the Latin adjective for edible, denoting its delicacy.

*Material examined*: *Holotype male*. INDIA: Arunachal Pradesh, Nimte, leg. Nikhil Joshi, 19.x.2018, 27.231°N, 93.552°E, 775 m.

*Paratypes*. Same collecting data as holotype; 3 M, 7 F.

*Diagnosis*. Dorsally bronzy black, antennae black, except fifth segment which is ochraceous with black base. Legs dark brown, mid- and hindcoxae are pale brown. Lateral margins of connexivum black. Ventrally rostrum, pleural area and last two segments of abdomen are blackish brown. Mid- and hind-coxae pale brown. Second to fourth abdominal segments brown (Figs 5C and 6C).

*Description*. Head transverse, 1.32 times wider than longer. Lateral margins in front of eye distinctly sinuate so that apex of head looks rounded (Fig 9C). Mandibular plates longer than clypeus, coarsely punctate. Clypeus transversely ridged. Eyes moderately large, pedunculate. Ocelli prominently closer to eyes than to each other; interocellar distance 1.61 times distance between eyes and ocelli. Antennae with scattered black setae especially on second to fourth segments and a few on fifth. First segment slightly surpassing apex of head, rounded, second, third and fourth laterally compressed, flattened with medial depression; fourth longest, slightly dilated beyond middle, fifth segment fusiform. Labium four segmented, first segment reaching base of head, second segment longest reaching base of fore coxae, third and fourth segment equal in length with fine setae.

Pronotum rhomboidal; anterior margin medially rounded behind head, anterior angles obtuse, lateral margins straight, raised as carinae; surface evenly punctate and rugulose; callus smooth, rugulose without punctures. Humeral angles rounded, faintly raised dorsally. Prosternum deeply punctate mesially. Mesosternum medially sulcate with two broad shining smooth patches on either side of midline. Metasternum narrow with a medial sulcus between metacoxae.

Scutellum punctate, apex broadly rounded.

Hemelytra. Clavus broad at base, narrowed distally and shorter than scutellum; corium broad, finely rugulose punctate. Membrane broad, with a series of basal cells and many parallel longitudinal veins. Hemelytra surpasses abdominal apex.

Legs. Femora laterally compressed. Tibiae sulcate with numerous spines. Tarsi well developed, first and third segments equal with long setae, middle shortest; first segment with dense setae underneath, claws and pulvilli well developed; claws divergent.

Abdomen very finely rugulose, though appear smooth, slightly punctate medially. Seventh sternum longest, posterior third rugulose, covering convex ventral portion of pygophore. Exposed portion of pygophore finely rugulose. Spiracles prominent. Trichobothria as usual for the genus with a pair posterior to spiracles.

Male genitalia. Pygophore black, posterior half more sclerotized than anterior, moderately convex ventrally (Fig 11C). Ventral rim slightly convex, denticulate (Fig 10C), dorsal rim rounded, with punctures. Parameres short, highly sclerotized, rugose, broad and apically rounded, outer margin slightly serrated and setose apically, inner margin straight (Fig 12C) and without a basal lobe.

*Geographical distribution*. Presently known only from Arunachal Pradesh, India.

*Remarks*. This species is a delicacy among the ethnic communities. Consumption in large quantities can be neurotoxic, and the person becomes photophobic, such as wanting to hide under the carpet or cot; or, as the people believe, ‘they begin to act like the bug, which hides under the stones or goes into cracks. If medical treatment is not sought, this behaviour can remain for a longer period, like for many months or even longer (personal communication with native people). The chemicals secreted by the bugs through the metathoracic glands might be the reason for intoxication.

**Fig 7:**
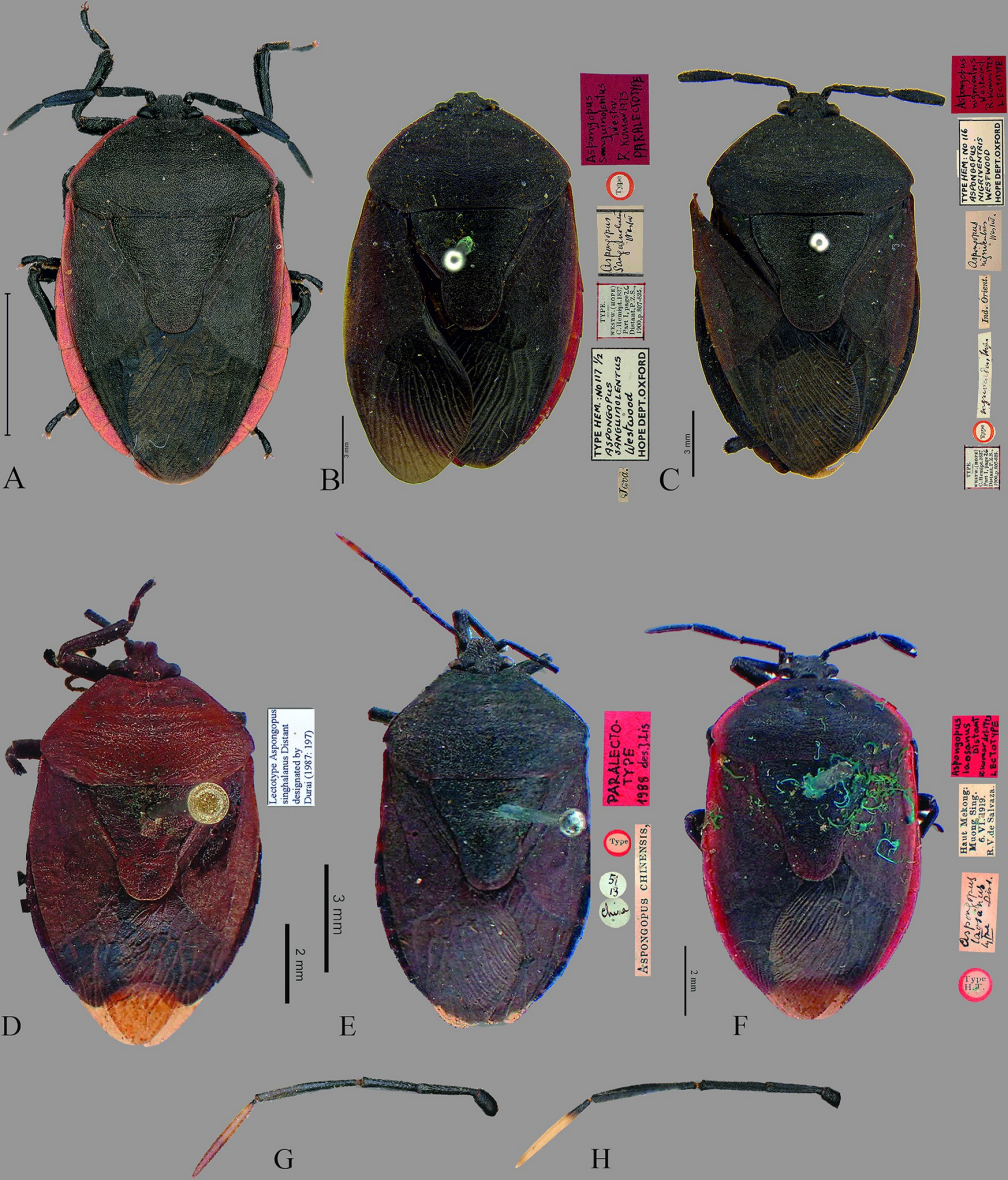
Dorsal habitus of *Coridius* spp. (A) *C*. *laosanus*. (B) *C*. *sanguinolentus* (Paralectotype). (C) *C*. *nigriventris* (Lectotype). (D) *C*. *singhalanus* (Lectotype). (E) *C*. *chinensis* (Paralectotype). (F) *C*. *laosanus* (Lectotype). (G) Antenna of *C*. *esculentus* **sp. nov.,** (H) Antenna of *C*. *chinensis*.

**Fig 8:**
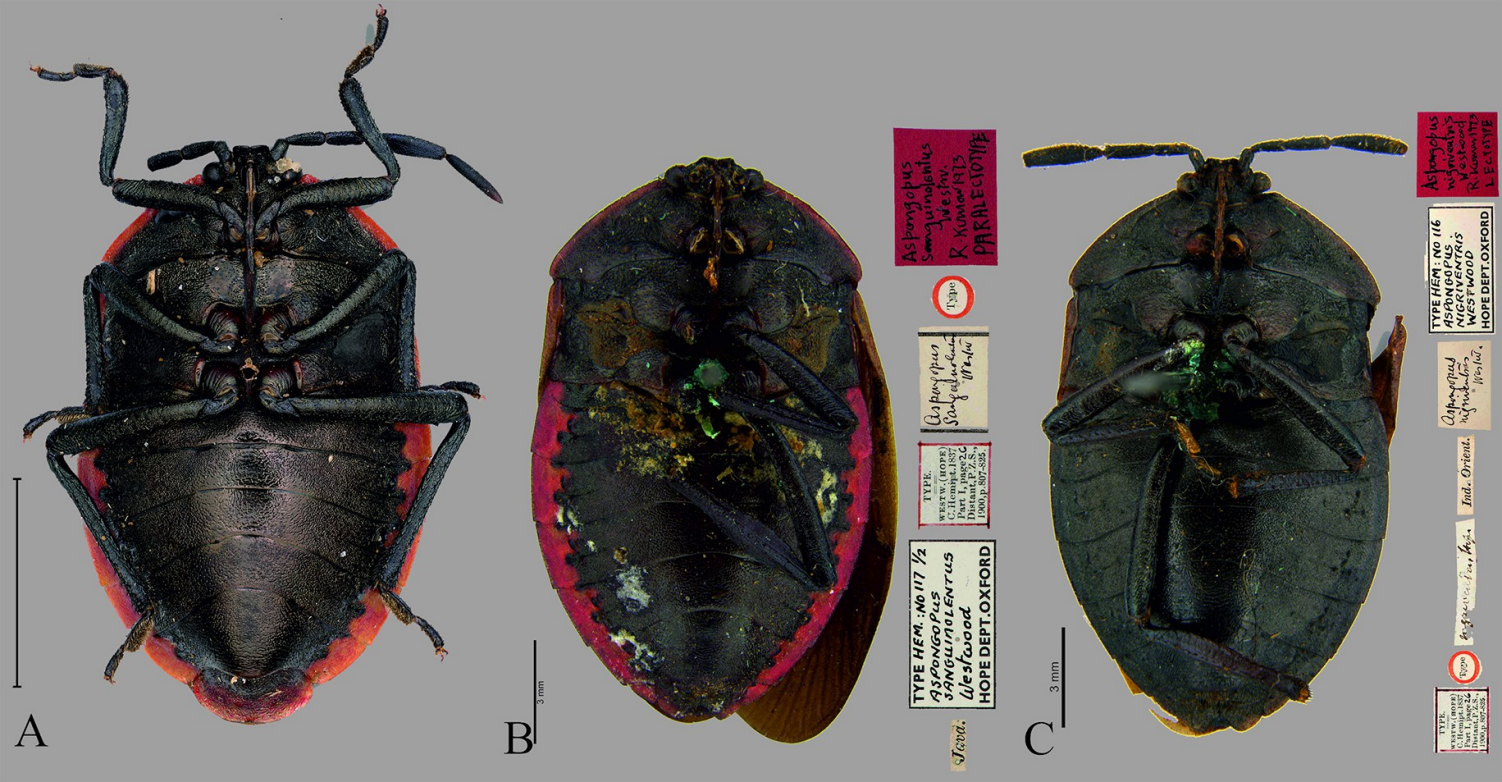
Ventral habitus of *Coridius* spp. (A) *C*. *laosanus*. (B) *C*. *sanguinolentus* (Paralectotype). (C) *C*. *nigriventris* (Lectotype).

**Fig 9:**
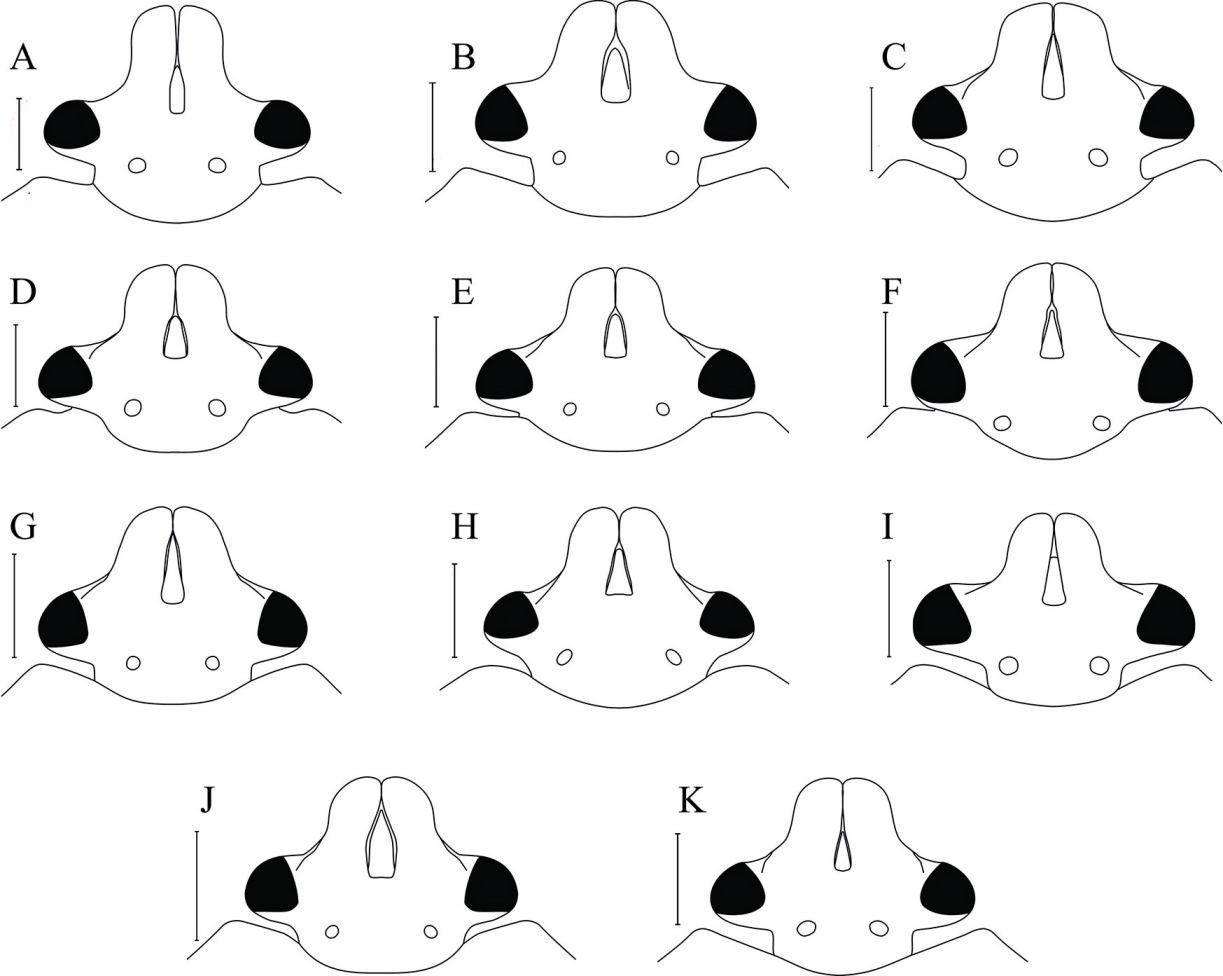
Dorsal view of head. (A) *C*. *insperatus* **sp. nov.,** (B) *C*. *adii* **sp. nov.,** (C) *C*. *esculentus* **sp. nov.,** (D) *C*. *nepalensis*. (E) *C*. *singhalanus*. (F) *C*. *brunneus*. (G) *C*. *assamensis*. (H) *C*. *ianus*. (I) *C*. *fuscus*. (J) *C*. *laosanus*. (K) *C*. *chinensis*. (Scale bar = 1 mm).

***Coridius laosanus* (Distant, 1921)** (Figs 7A and 7F, 8A, 9J).

*Aspongopus laosanus* Distant, 1921: 167; Hoffmann, 1935: 119.

*Coridius laosanus* Yang, 1940: 7; Durai, 1987: 188, 199, 200, Figs 184, 185; Lis, 1990: 141; 1992: 38; Kocorek, 2003: 51, 55.

Material examined (images): *Lectotype male*: *Aspongopus laosanus* Distant, R. Kumar det. 1973, LECTOTYPE // Type H. T. // Haut Mekong: Muong Sing, 6. VI. 1919., R. V. de Salvaza. // *Aspongopus laosanus* type Dist.

*Material examined*. NE LAOS: Houaphanh Province, Mt. Phu Pane, 27.12°N, 103.58°E, 22. V. 2011, 1200–1600 m. Donated by Dr. Marcos Roca-Cusachs.

*Diagnosis*. Head, antennae, and rostrum black; pronotum blackish brown with reddish orange stripe along lateral margins; connexivum completely reddish orange; scutellum concolorous with pronotum disk; corium black with basal third reddish orange; costal margin extending along connexivum and membrane black (Fig 7A). Legs blackish brown. Venter blackish brown, with reddish orange stripe along lateral margins.

Head wider than long, mandibular plates longer than clypeus, rounded anteriorly and meeting in front of clypeus (Fig 9J). Eyes globular, pedunculate. Ocelli well developed, closer to the eyes than to each other; interocellar distance 1.75 times distance of between eyes and ocelli (Fig 9J). Labium four segmented, reaching anterior margin of meso coxae. First segment extending beyond head.

*Geographical distribution*. China, India, Laos, Thailand, and Vietnam [24, 27, 42].

*Remarks*. This species was described from Laos and is known only from its type locality. Lis [24] mentioned that this species is present in India without any further information. In their world catalogue, Rolston et. al., [42] also mentioned China and Vietnam. Later, Kocorek [43] reported this species for the first time from Thailand. The specimens used here were donated by Marcos Roca-Cusachs, which were collected from the Laos and are identical to the holotype, as observed from images (Fig 7A and 7F).

***Coridius sanguinolentus* (Westwood, 1837)** (Figs 7B and 8B) (Paralectoype images).

*Aspongopus sanguinolentus* Westwood, 1837: 26; Dallas, 1851: 350; Walker, 1868: 483; Stål, 1870: 85; Atkinson, 1889: 89, 90; Lethierry & Severin, 1893: 238; Distant, 1901: 819; 1902: 284; Kirkaldy, 19090: 257; Hoffmann, 1932a: 9; 1932b: 140; Wu, 1933: 216; Chatterjee, 1934: 27; Hoffmann, 1935: 120; Yang, 1962: 46, 49, pl. 4, Fig 32; Zhang, 1985: 54, pl. 47, Fig 159; Hua, 1989: 43.

*Aspongobus* [sic] *sanguinolentus* Dohrn, 1859: 21.

*Aspongopus circumcinctus* Walker, 1868: 483; Atkinson, 1889: 91; Lethierry & Severin, 1893: 236; Distant, 1900: 233; 1901: 104. Synonymized by Distant, 1902: 284.

*Aspongopus* (*Aspongopus*) *sanguinolentus* Schouteden, 1913: 9; Tang, 1935: 356.

*Coridius sanguinolentus* Yang, 1940: 10, 22, 23, Fig 7; Hoffmann, 1948: 23; Stichel, 1962a: 725; 1962b: 205; Durai, 1986: 6; 1987: 188, 206, Figs 212, 213; Lis, 1990: 115, 142.

Material examined (images): *Paralectotype Female*: INDONESIA: Java. Labels: TYPE HEM.: No 117 ½, *Aspongopus sanguinolentus* Westwood, HOPE DEPT.OXFORD // “TYPE., WESTW." (HOPE), C. Hemipt. 1837, Part I, page 26, Distant, P.Z.S., 1900, p. 807–825, // *aspongopus sanguinolentus* Westw // Type // *Aspongopus sanguinolentus* Westw., R. Kumar 1973, PARALECTOTYPE.

*Diagnosis*. Entirely reddish brown with connexivum and a broad lateral abdominal patch reddish-ochraceous (Fig 8B). Head triangular, mandibular plates longer than clypeus. Ocelli reddish brown. Antennae black, five segmented, first segment surpassing apex of head, second and third subequal, fourth longest. Pronotum declivous, lateral margins rounded raised and reddish in color, anterior margin concave, posterior margin straight. Scutellum broad, apically rounded. Femora inwardly curved with spines.

*Geographical distribution*: China, India (Assam and Uttarakhand), Indonesia, Myanmar, Vietnam [24, 27, 42].

***Coridius nigriventris* (Westwood, 1837)** (Figs 7C and 8C) (Lectotype images).

*Aspongopus nigriventris* Westwood, 1837: 26; Dallas, 1851: 349; Vollenhoven, 1868: 39; Walker, 1868: 482; Stål, 1870: 85; Distant, 1879: 45; Atkinson, 1889: 89; Kirby, 1892: 88; Lethierry & Severin, 1893: 237; Breddin, 1900: 331, 332; Distant, 1901: 819; 1902: 284; Kirkaldy, 1909: 256; Schumacher, 1917: 446; Distant, 1921: 166; Hoffmann, 1935: 120; Villiers, 1952: 85; Yang, 1962: 48; Hsiao *et al*., 1977: 70, pl. 8, Fig 122; Datta *et al*., 1985: 5, Fig 8; Zhang & Lin, 1988: 85; Hua, 1989: 43.

*Aspongobus* [sic] *nigriventris* Dohrn, 1859: 21.

*Aspongopus* (*Aspongopus*) *nigriventris* Schouteden, 1913: 8.

*Coridius nigriventris* Yang, 1940: 7, 10, 21, 22, Fig 6; Hoffmann, 1948: 23; Schaefer & Ahmad, 1987: 30; Zhang & Lin, 1987: 76.

*Coridius nepalensis* Durai, 1987: 198; Rolston *et al*., 1996: 42, 43, 100.

Material examined (images): *Lectotype female*. INDIA: Labels: TYPE WESTW. (HOPE), C. Hemipt. 1837, Part I, page 26, Distant, P.Z.S., 1900, p. 807–825 // Type // nigriventris, West // Ind. Orient. // *aspongopus nigriventris* Westw. // TYPE HEM: No. 116, *Aspongopus nigriventris*, Westwood, HOPE DEPT. OXFORD *// Aspongopus nigriventris* Westwood, R. Kumar 1973, LECTOTYPE.

*Diagnosis*. Entire body blackish brown except coria and ocelli which are paler. Connexivum without yellow spots in between segmentation. Body oval, lateral margin of pronotum rounded; anterior margin slightly concave and posterior margin rounded; lateral margins raised and brown in colour. Antennae five segmented, first segment surpasses apex of head, second 0.84 times longer than third, fourth longest and laterally compressed. Femora slightly curved inwards. First segment of tarsus densely setose, second and third with very sparse long golden setae.

*Geographical distribution*. India (Kerala and Maharashtra) [42].

*Remarks*. This species was described as *Aspongopus nigriventris* (= *Coridius nigriventris*) by Westwood in 1837 from India. The exact type locality was not mentioned in the original description. Durai synonymized this species with *C*. *nepalensis* in 1987, considering the similarity of the aedeagus with the latter. However, in this study, we used the sequence deposited as *C*. *nigriventris* (accession #JQ387600) in GenBank for molecular analysis. We found that *C*. *nigriventris* and *C*. *nepalensis* form separate lineages (see Fig 4). Additionally, upon comparing type images, we noted some morphological differences. Therefore, we reinstate the status of *C*. *nigriventris* as a valid species.

**Fig 10:**
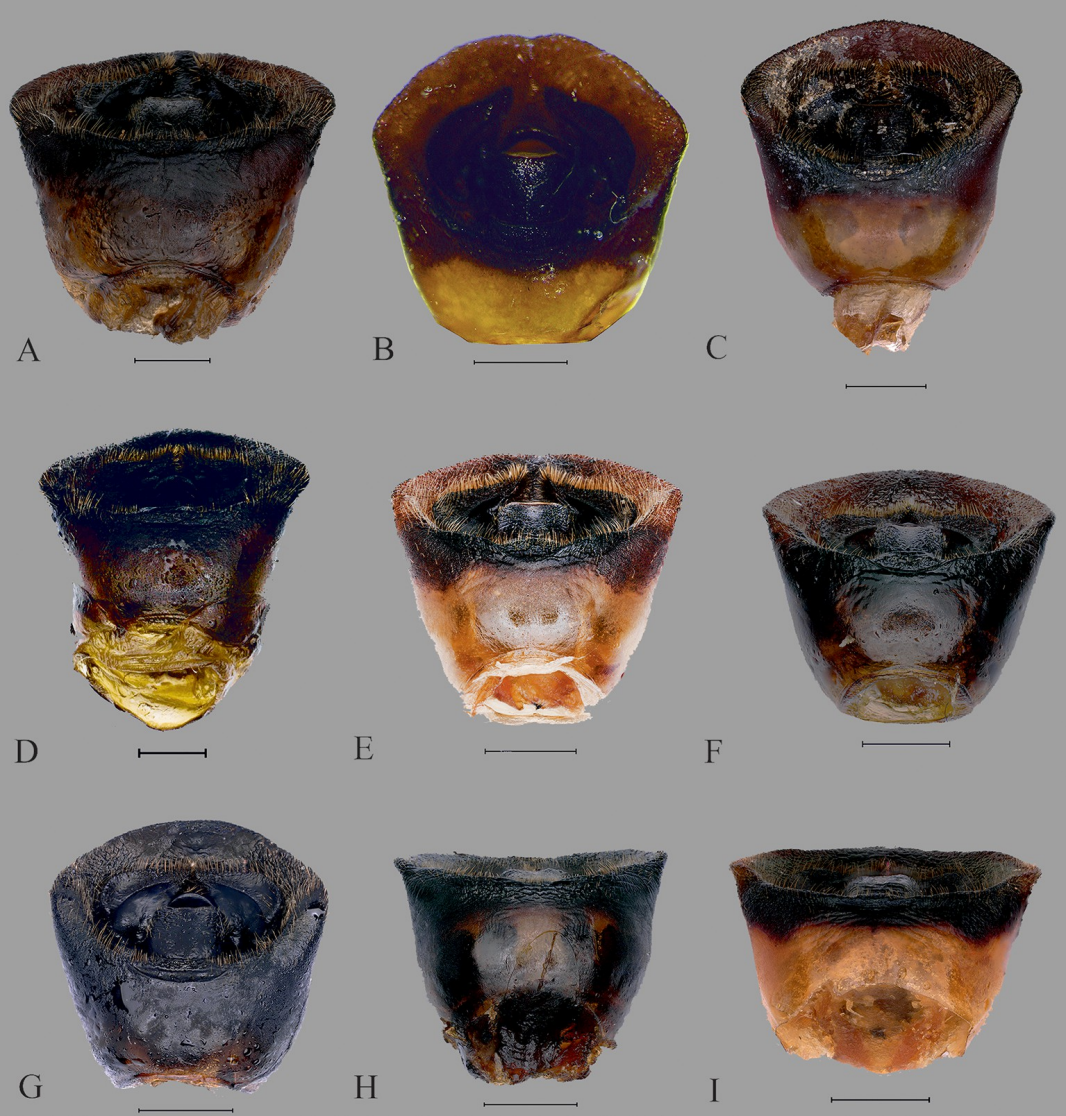
Dorsal view of male genitalia. (A) *C*. *insperatus* **sp. nov.,** (B) *C*. *adii* **sp. nov.,** (C) *C*. *esculentus* **sp. nov.,** (D) *C*. *nepalensis*. (E) *C*. *singhalanus*. (F). *C*. *brunneus* (G) *C*. *assamensis*. (H) *C*. *ianus*. (I) *C*. *fuscus*. (Scale bar = 1 mm).

**Fig 11:**
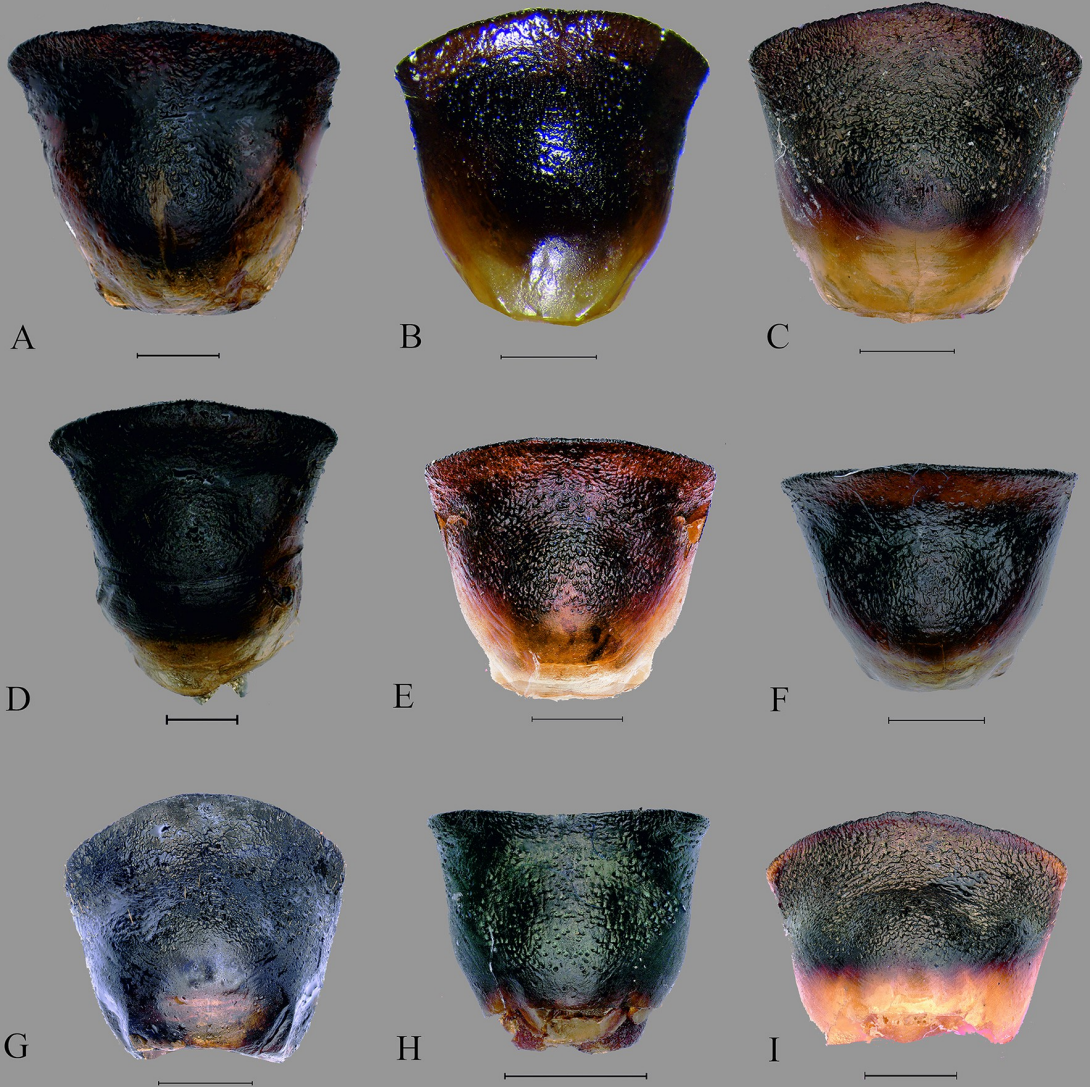
Ventral views of male genitalia. (A) *C*. *insperatus* **sp. nov.,** (B) *C*. *adii* **sp. nov.,** (C) *C*. *esculentus* **sp. nov.,** (D) *C*. *nepalensis*. (E) *C*. *singhalanus*. (F) *C*. *brunneus*. (G) *C*. *assamensis*. (H) *C*. *ianus*. (I) *C*. *fuscus*. (Scale bar = 1 mm).

**Fig 12:**
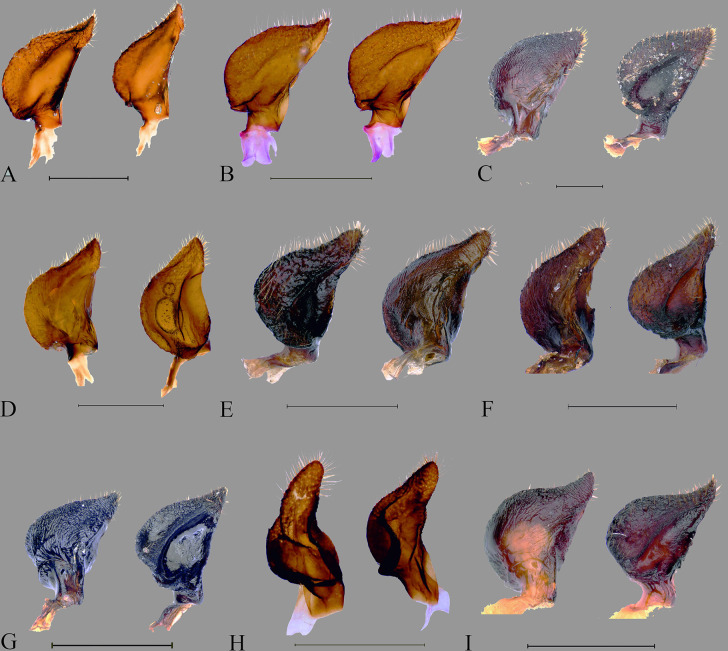
Parameres of *Coridius* spp. in various views: left paramere in dorsal, and right paramere in ventral. (A) *C*. *insperatus* **sp. nov.,** (B) *C*. *adii* **sp. nov.,** (C) *C*. *esculentus* **sp. nov.,** (D) *C*. *nepalensis*. (E) *C*. *singhalanus*. (F) *C*. *brunneus*. (G) *C*. *assamensis*. (H) *C*. *ianus*. (I) *C*. *fuscus*. (Scale bar = 1 mm).

***Coridius nepalensis* (Westwood, 1837)** (Figs 5D, 6D, 9D, 10D, 11D and 12D).

*Aspongopus nepalensis* Westwood, 1837: 26; Dallas, 1851: 349; Walker, 1868: 483; Stål, 1870: 85; Distant, 1879: 45; Atkinson, 1889: 90; Lethierry & Severin, 1893: 237; Waterhouse, 1900a: 251; 1900b: 9; Distant, 1901a: 819; 1901b: 104; 1902: 283; Kirkaldy, 1909: 256; Maxwell-Lefroy, 1909: 678; Strickland, 1932: 873–875; Miller, 1956: 38.

*Aspongobus* [sic] *nepalensis* Dohrn, 1859: 21.

*Coridius nepalensis* Yang, 1940: 7, 8; Durai, 1987: 190, 198, 265; Lis, 1990: 114, 141; 1992: 37; Rolston *et al*., 1996: 42, 43, 99; Ahmad *et al*., 1997: 307, 310, 319.

*Material examined*. INDIA: Arunachal Pradesh, Tippi, leg. Thejavikho Chase, 30.xi.2020, 27.026°N, 92.610°E, 179 m: 2 M, 2 F.

*Diagnosis*. Dorsally brown to dark brown, ventrally pale brown except abdominal sternites, seventh abdominal segment dark brown. Antennae black except fifth segment, which is pale ochraceous with fuscous apex. Lateral margins of pronotum black; area near metathoracic scent gland dark brown; legs concolorous with body (Figs 5D and 6D).

Head two times wider at eyes than longer, equal to the width of anterior pronotal margin. Mandibular plates smooth, much longer than clypeus and meeting in front of it, lateral margins sinuate, and head rounded at apex; clypeus faintly ridged mesially (Fig 9D). Eyes moderately large, pedunculate. Ocelli prominent, rounded, interocellar distance 1.97 times distance between eyes and ocelli. Antennae five segmented, all segments with fine sparse setae, fifth segment pilose. First segment surpassing apex of head, second segment 0.71 times shorter than third, fourth segment longest, fifth segment fusiform in shape. Labium four segmented, reaching to mid coxa; first segment reaching to base of head, second longest, third and fourth together equal to second.

Pronotum slightly declivous, deeply punctate, and medially transversely rugulose, anterior margin concave behind head, anterior angles obtuse, lateral margins appear carinated, posterior margin straight; calli prominent without rugae. Prosternum sulcate in the middle and laterally rugulose. Meso and meta sternum narrow with shallow groove.

Scutellum sparsely punctate and rugulose, apex tongue shaped.

Hemelytra. Corium finely rugulose, membrane broad with many veins.

Legs. Coxae rounded; femora flattened and slightly curved inside with short, stout, sparsely distributed setae; tibiae sulcate with numerous short spines like setae on both sides; tarsi with golden pubescence underneath, claws divergent.

Abdomen finely rugulose, glossy, without setae, seventh segment longest, proximal third with transverse rugae, distal two thirds smooth. Trichobothria situated posteriorly to spiracles.

Male genitalia. Exposed region of pygophore declivous basally, flat in distal half and with very sparse long setae. Elongate, somewhat squarish and sclerotized; ventral rim not rounded and with numerous long brown setae (Fig 10D); ventrally convex, punctate and without setae (Fig 11D). Parameres short, sclerotized, medially rounded, and broad and apically narrowing; outer margin irregular and appears denticulate and setose apically; inner margin sometimes a little concave (Fig 12D).

*Geographical distribution*. Bangladesh, India (Assam, and Arunachal Pradesh), Indonesia, Nepal, and Vietnam [24, 27, 42].

*Remarks*. *C*. *nepalensis* is found under stones in dry riverbeds. This species is commonly consumed by the Nyishi tribe and is harvested from the Dibang River’s riverbeds (Loklung village in Lower Dibang Valley, Arunachal Pradesh).

***Coridius singhalanus* (Distant, 1900)** (Figs 5E, 6E, 7D, 9E, 10E, 11E and 12E).

*Aspongopus singhalanus* Distant, 1900: 222; 1902: 283; Bergroth, 1908: 188.

Kirkaldy, 1909: 257; Gravely, 1915: 509.

*Coridius singhalanus* Durai, 1986: 6; Mathew, 1986: 43; Durai, 1987: 196; Lis, 1990: 113, 142; 1992: 37; Rolston et al., 1996: 48, 100; Ahmad et al., 1997: 307, 319.

*Material examined* (images): *Lectotype male*: *Aspongopus singhalanus* Distant, designated by Durai (1987: 197) (Fig 7D).

INDIA Assam, Sivsagar, leg. Anandita, 28.vii.2020, 26.982°N, 94.642°E, 100 m: 2 M, 3 F.

*Diagnosis*. Overall yellowish dorsally except head. Legs, membrane and connexivum fuscous. Lateral margins of mandibular plates, area near ocelli and eyes black; first four antennal segments black, fifth ochraceous with fuscous base and apex. Ocelli, callar region and base of the first antennal segment pale in colour. Basal fourth of clavus fuscous. Lateral margin of connexivum black, pale ventrally; rostrum, sternal area, coxae and pygophore ventrally pale; rest of body dark brown (Figs 5E and 6E).

Head transverse: mandibular plates longer than clypeus, meeting in front of it and apex rounded, lateral margins sinuate (Fig 9E), with sparse punctures; callar area distinct without punctures. Eyes pedunculate. Ocelli well developed, rounded, closer to eye than to each other. Antennae five segmented, first segment surpassing apex of head, second and third sub equal in length, fourth longest; second, third and fourth segments sulcate and laterally compressed, fifth finely setose and fusiform. Labium four segmented, reaching middle of mesosternum; first and second labial segments equal in length, third and fourth short. Bucculae prominent.

Male genitalia. Pygophore proximally narrow and distally broad, sclerotized; ventral rim of the pygophore slightly depressed with setae (Fig 10E); moderately convex ventrally, with punctures and without setae (Fig 11E). Parameres sclerotized with rugose, medially broad and rounded, apex blunt, outer margin serrated and setose apically, inner margin moderately concave (Fig 12E).

*Geographical distribution*: India (Assam and Arunachal Pradesh), Indonesia, and Sri Lanka [24, 27, 42, 44].

*Remarks*: Presence of this species in India was mentioned by Lis [24] without giving any illustrations.

***Coridius brunneus* (Thunberg, 1783)** (Figs 5F, 6F, 9F, 10F, 11F and 12F).

*Cimex brunneus* Thunberg, 17 83: 45.

*Cimex obscurus* Fabricius, 1794: 107. Synonymized by Durai, 1987: 195.

*Cimex obscurior* Turton, 1802: 642.

*Edessa obscura* Fabricius, 1803: 151, Wolff, 1811: 173, Fig 17.

*Edessa* (*Dinidor*) *obscura* Latreille, 1829: 195.

*Aspongopus obscurus* Burmeister, 1835: 352; Westwood, 1837: 6; Herrich-Schäffer, 1844: 80; Dallas, 1851: 349; Herrich-Schäffer, 1851: 307; Stål, 1868: 38; Vollenhoven, 1868: 38; Walker, 1868: 482; Atkinson, 1889: 88; Sharp, 1890: 404; Kirby, 1892: 88; Lethierry & Severin, 1893: 238; Breddin, 1899: 168; 1901: 14, 63; Distant, 1902: 283; Maxwell-Lefroy, 1909: 313; Behura et al., 1972: 88, 104; Behura *et al*., 1977: 113–128; Hsiao *et al*., 1977: 70, pl. 8, Fig 125; Verma *et al*., 1979: 873–878.

*Spongopodium obscurus* Spinola, 1837: 305, 306.

*Aspongopus ochreus* Westwood, 1837: 6, 25; Vollenhoven, 1868: 38, 39; Walker, 1868: 483; Stål, 1870: 85; Distant, 1879: 45, 52; Atkinson, 1889: 89; Lethierry & Severin, 1893: 238; Distant, 1901: 819; 1902: 283; Kirkaldy, 1909: 257. Synonymized by Lis, 1990: 113.

*Aspongobus* [sic] *ochreus* Dohrn, 1859: 21.

*Aspongobus* [sic] *obscurus* Dohrn, 1859: 21.

*Dinidor obscura* Carpenter & Westwood, 1863: 564

*Aspongopus brunneus* Mayr, 1866: 69; Distant, 1879: 45, 52; Atkinson, 1889: 87; Lethierry & Severin, 1893: 236; Distant, 1901: 104; 1902: 282, 283; Kirkaldy, 1909: 255; Maxwell-Lefroy, 1909: 678; Distant, 1921: 166; Fletcher, 1921: 189, 190; Misra, 1923: 301; China, 1928: 195, 196; Hoffmann, 1935: 118; Yang, 1962: 47, 48; Zhang & Lin, 1988: 84; Zheng & Jin, 1990: 142.

*Aspongopus* (*Aspongopus*) *brunneus* Stål, 1870: 82; Schouteden, 1913: 7.

*Aspongopus* (*Aspongopus*) *obscurus* Stål, 1868: 82.

*Aspongopus orientalis* Kirkaldy, 1909: 257; Manna, 1951: 40, 41, 44, Figs 20a-h.

*Aspongopus* (*Aspongopus*) *ochreus* Schouteden, 1913: 8.

*Aspongopus* (*Aspongopus*) *orientalis* Schouteden, 1913: 8, pl. 1, Fig 3.

*Coridius ochreus* Yang, 1940: 8; Durai, 1986: 6; 1987: 190, 196, Figs 171–172; Ahmad *et al*., 1997: 307, 319.

*Coridius orientalis* Nuamah, 1982: 16.

*Coridius brunneus* Durai, 1986: 5; 1987: 109, 195, 196, Figs 161–170; Schaefer & Ahmad, 1987: 30; Zhang & Lin, 1987: 76; Lis, 1990: 113, 140; 1992: 37; Rolston *et al*., 1996: 33–35, 98; Ahmad *et al*., 1997: 308, 315, 319, 320.

*Coridius obscurus* Senrayan & Annadurai, 1991: 237–243, Figs a-c.

*Material examined*. INDIA: Maharashtra, Pune, leg. Swapnil Boyane, 12.i.2019, 18.653°N, 73.909°E, 550 m: 1 F.—INDIA: Maharashtra: Khopoli, leg. Hemant Ghate, 23.x.2015, 18.793°N, 73.334°E, 69 m: 1 M.

*Diagnosis*. Overall body yellowish brown to dark brown; pale yellow ventrally, brown in areas near coxae and sternum rest dark brown. Lateral margins of pronotum not black. Antennae with first segment basally ochraceous, distally dark brown, second and third black, fourth ochraceous except a minute region at base and apex which are black (Figs 5F and 6F). Legs brown to blackish brown, tibia slightly darker.

*Redescription*. Overall body sparsely finely punctate, almost smooth medially underneath, and very finely punctate laterally. Abdomen finely rugulose and punctate.

Head rounded at apex, with mandibular plates much longer than clypeus (Fig 9F); eyes large, bulbous, silvery in colour. Ocelli yellowish brown placed closer to eye than to each other. Labium four segmented reaching middle of mesosternum. Antennae five segmented, first segment reaching apex of head, fifth segment blackish brown.

Pronotum glossy and finely punctate, anterior border deeply concave, lateral margins curved, posterior margin straight; calli smooth, shining with sparse punctures. Pro-sternum with a distinct elevated smooth region on either side of the median groove, laterally rugulose and coarsely punctate. Meso and meta sternum smooth medially, rugulose and coarsely punctate in pleural regions. Femora with spines and setae underneath, tibae distinctly sulcate and spined ventrally as well as dorsally, first segment of tarsus densely setose, second and third with very sparse long setae; claws widely separated, pulvilli distinct, well developed.

Scutellum long, rugulose punctate in basal two third; reaching half-length of abdomen, apex rounded.

Male genitalia. Pygophore proximally narrow and distally slightly broad, sclerotized ventrally convex, with deep punctures (Fig 11F); ventral rim rounded with sparse setae (Fig 10F), parameres curved, highly sclerotized with rugae, outwardly remarkably rounded and its tip narrowed, serrated, and setose apically, inner margin moderately concave (Fig 12F).

*Geographical distribution*: Asutralia, Borneo, China, India, Indonesia, Malaysia, Myanmar, Sri Lanka [24, 27, 42, 44].

***Coridius assamensis* (Distant, 1902)** (Figs 5G, 6G, 9G, 10G, 11G and 12G).

*Aspongopus assamensis* Distant, 1902: 285; Bergroth, 1908: 188; Kirkaldy, 1909: 255, Chen, 1983: 45, 46; Datta *et al*., 1985: 4, Fig 7; Chen & Yang, 1988: 90.

*Aspongopus* (*Aspongopus*) *assamensis* Schouteden, 1913: 7.

*Coridius assamensis* (Distant), Durai, 1987: 190, 198, 199, Figs 182–183; Lis, 1990: 114, 140; 1992: 37; Rolston *et al*., 1996: 33, 98; Ahmad *et al*., 1997: 307; 319; Kocorek, 2003: 51, 55.

*Material examined*. Lectotype male (Images): *Aspongopus assamensis* Dist., R. Kumar det. 1973, LECTOTYPE // Distant Coll. 1911–383. // assamensis Dist // LECTOTYPE.

INDIA: Arunachal Pradesh, Loklung, leg. Gumnya Ete, 2.i.2019, 27.960°N, 95.566°E,139 m: 1 M.

*Diagnosis*. Entire body including antennae piceous. Eyes and ocelli dark brown (Figs 5G and 6G). Metathoracic scent glands opening area matte black.

*Redescription*. Head 1.86 times wider than longer, deeply punctate, mandibular plates almost two times longer than clypeus and meeting in front of it; apex of head rounded, lateral margin deeply sinuated (Fig 9G), lateral border of mandibular plates and area in front of ocelli smooth. Eyes, large, pedunculate. Ocelli rounded; interocular distance almost twice distance between ocelli and eyes. Antennae five segmented, first segment passing apex of head, second segment 0.70 times longer than third, fourth segment longest, fifth segment 0.44 times smaller than fourth. All segments with sparsely distributed setae, second, third and fourth segments compressed, fifth fusiform.

Pronotum declivous, trapezoid, with densely packed deep punctures, lateral margins smooth and raised as carina; anterior margin concave behind head, anterior angles blunt; posterior angles obtuse, posterior margin of pronotum more or less straight; calli prominent, smooth and without punctures.

Scutellum with punctures like pronotal, apex flat and tongue shaped.

Hemelytra. Clavus short, narrow; corium with fine punctures, rugulos. Membrane darker than corium and with many longitudinal veins. Hemelytra surpassing apex of abdomen.

Legs. Coxae rounded; femora slightly inwardly curved, laterally compressed with a series of small stout spinules and sparse long setae; tibiae almost straight and with numerous series of spines on both sides; tarsus with long golden pubescence underneath, pretarsus with few long setae; claws well developed and divergent.

Abdomen very finely rugulose, median region slightly less rugulose and smooth, seventh sternum longest, rugulose in posterior third, setose at posterior border; Connexivum exposed with pale yellow patch in between segmentation. Spiracles rounded, prominent, closer to anterior border than to lateral border of respective segment. Trichobothria paired, posterior to spiracles.

Male genitalia. Pygophore, squarish, exposed portion finely rugulose; moderately convex ventrally, with punctures and few sparse setae (Fig 11G). Ventral rim rounded with numerous long brown setae (Fig 10G). Parameres glossy, highly sclerotized with rugae, short, medially broad, and rounded, apically narrowing. Outer margin of paramere serrated and setose apically, inner margin more or less straight (Fig 12G).

*Geographical distribution*: China, India (Assam and Arunachal Pradesh), Nepal, and Vietnam [24, 27, 44].

*Remarks*: *C*. *assamensis* is often collected from the riverbeds in Dibang valley and consumed by the indigenous communities especially by the Nyishi tribe.

***Coridius ianus* (Fabricius, 1775)** (Figs 5H, 6H, 9H, 10H, 11H and 12H).

*Cimex ianus* Fabricius, 1775: 714; 1781: 357; 1787: 295.

*Cimex janus* Fuessly, 1778: 163; Goeze, 1778: 247; Gmelin, 1790: 2152.

*Cimex surinamensis* Goeze, 1778: 232; Gmelin, 1790: 2134. Synonymized by Atkinson, 1889.

*Cimex afer* Drury, 1782: 66, 67, pl. 46, Fig 7. Synonymized by Fabricius, 1794: 107.

*Cimex danus* [sic] Fabricius, 1794: 107; Wolff, 1800: 13, pl. 2, Fig 13^13^.

*Cimex surinamensis* Gmelin, 1789: 2134. Synonymized by Atkinson, 1889: 88.

*Edessa ianus* Fabricius, 1803: 151.

*Coridius ianus* llliger, 1807: 361.

*Pentatoma janus* Lepeletier & Serville, 1825: 56.

*Aspongopus janus* Laporte, 1833: 58; Burmeister, 1835: 352; Westwood, 1837: 6; Amyot & Serville, 1843: 173, 174; Herrich-Schäffer, 1844: 78, Fig 747; Dallas, 1851:348; Dohrn, 1860: 401; Mayr, 1866: 70; Walker, 1868: 482; Distant, 1879: 45; Atkinson, 1889: 88, 89; Kirby, 1892: 88; Lethierry & Severin, 1893: 237; Distant, 8331901a: 823; 1902b: 104; 1902: 281, 282, Fig 179; Maxwell-Lefroy, 1909: 313; Mann, 1911: 1166, 1167, 2 Figs; Fletcher, 1917: 57, 289; 1921: 189; Singh-Pruthi, 1925: 148, pl. 9, Fig 27; Ayyar, 1933: 17, Fig 14; Chatterjee, 1934: 27; Kathuria *et al*., 1975: 31–33; Behura & Das, 1977: 49–60; Behura *et al*., 1978: 55–66; Dhiman, 1981: 180, 181; Singh & Narain, 1984: 259–267; Mishra & Sharma, 1990: 480, 481; Sharma, 1990: 1329, 1330.

*Aspongopus vicinus* Westwood, 1837: 25. Synonymized by Dallas, 1851: 348.

*Aspongobus* [sic] *janus* Dohrn, 1859: 21.

*Aspangopus* [sic] *janus* Motschoulsky, 1863: 76.

*Aspongopus* (*Aspongopus*) janus Stål, 1870: 82, 83; Schouteden, 1913: 8, pl. 1, Fig 8.

*Aspongopus ianus* Breddin, 1909: 282; Kirkaldy, 1909: 380; Maxwell-Lefroy, 1909: 678, pl. 74, Fig 3; Manna, 1951: 39, 40, 44, Figs 19 a-i.

*Coridius janus* Schumacher, 1924: 335, 336; Scudder, 1959: 413; Kumar, 1962: 48, 51, 53–56, Figs 25–27, 46, 47, 72–74, 88; Rastogi & Kumari, 1962: 69–77; Gentry, 1965: 147, 154, 162, Fig 32; Ahmad & Abbasi, 1971: 37–49, Figs 1–14; Ahmad *et al*., 1971: 1–16; Naqvi *et al*., 1973: 209–221; Ahmad *et al*., 1974: 175–188, Figs 1–5; Ahmad & Afzal, 1977: 1–4, Figs 1–4; Kaushik *et al*., 1977: 244–250; Kaushik i, 1979: 211–219; Ahmad, 1980: 135; Nuamah, 1982: 16, 25; Schaefer & Ahmad, 1987: 30; Afzal & Sahibzada, 1988: 254; Satapathy & Patnaik, 1991: 56, 59, 60, Tables 1 and 2, Fig 1G–1L.

*Coridius ianus* Durai, 1987: 187, 216–218; Lis, 1990: 122, 141, Fig 23; 1991: 84, 87, Fig 4; 1992: 38; Rolston *et al*., 1996: 40, 41, 99; Ahmad *et al*., 1997: 305–307, 310–313, 318, 319, Figs 2A–2G.

*Material examined*. INDIA: Arunachal Pradesh, Rani Village, Pasighat, leg. Nikhil Joshi, 1.x.2018, 27.9653°N, 95.316°E, 139 m: 1M.

*Diagnosis*. Head, antennae, callar region, anterior half of scutellum, membrane, and legs black; pronotum, clavus, corium, connexivum and posterior half of scutellum orange or red; abdomen blackish ventrally. Hemelytra, corium similar to pronotum but membrane black. Prosternal groove yellow. Legs dark brown to blackish. Abdomen ventrally almost sanguineous to black with a narrow lateral border which is pale brown (Figs 5H and 6H).

Head. Mandibular plates longer than clypeus, apex rounded (Fig 9H). Eyes large, bulbous. Ocelli placed closer to eye than to each other. Labium short, reaching base of procoxae. Antennae five segmented, first antennal segment reaching apex of head. Pronotum glossy, finely punctate; pronotum with concave anterior margin, sinuate lateral margins, straight posterior margin.

Scutellum long, its sides gently sinuate, apex rounded,

Male genitalia. Pygophore with ventral rim concave with sparse setae; dorsal bridge straight with long ochraceous setae (Fig 10H); ventrally moderately convex, with deep punctures (Fig 11H). Parameres curved, highly sclerotized with rugae, tip blunt; outer margin serrated and setose apically; inner margin moderately concave (Fig 12H).

*Geographical distribution*. Bangladesh, Borneo, India, Indonesia, Madagascar, Mynmar, Pakistan, Phillippienes, Sri Lanka, and Thailand [24, 27, 42, 44].

*Remarks*. This species is widely distributed in Indo-Malayan region.

***Coridius fuscus* (Westwood, 1837)** (Figs 5I, 6I, 9I, 10I, 11I and 12I).

*Aspongopus fuscus* Westwood, 1837: 26; Dallas, 1851: 349; Vollenhoven, 1868: 39; Walker, 1868: 483; Stål, 1870: 85; Stål, 1871: 609, 645; Atkinson, 1889: 90; Lethierry & Severin, 1893: 237; Breddin, 1900: 332; Distant, 1900: 221; 1901a: 819; 1901b: 104; 1902: 284, 285; Kirkaldy, 1909: 256; Distant, 1921: 166; Lehmann, 1923: 182; hina, 1928: 195; Hoffmann, 1931: 144; 1932: 1013; Schouteden, 1933: 53; Wu, 1933: 215, 216; Cheo, 1935: 28; Hoffmann, 1935: 119; Yang, 1962: 47, 48; Chang, 1974: 356; Hsiao *et al*., 1977: 70, pl. 9, Fig 124; Hua, 1989: 43.

*Aspongopus marginalis* Dallas, 1851: 350; Walker, 1868: 483; Stål, 1870: 85; Atkinson, 1889: 90, 91; Lethierry & Severin, 1893: 237. Synonymized by Distant, 1900: 221.

*Aspongobus* [sic] *fuscus* Dohrn, 1859: 21.

*Aspongobus* [sic] *marginalis* Dohr, 1859: 21.

*Aspongopus* (*Aspongopus*) *fuscus* Schouteden, 1913: 8; Tang, 1935: 356.

*Coridius fuscus* Yang, 1940: 8, 10, 19–21, Fig 5; Hoffmann, 1948: 22, 23; Stichel, 1962a: 725; 1962b: 205; Sienkiewicz, 1964: 112; Durai, 1986: 4, 5; 1987: 188, 202, 203, Figs 197–200; Schaefer & Ahmad, 1987: 30; Lis, 1990: 114, 115, 141; 1992: 38; Rolston *et al*., 1996: 39, 40, 99.

*Material examined*. INDIA: Maharashtra, Raigad, leg. Hemant Ghate, 1.x.2011, 18.771°N, 73.365°E, 70 m: 1 M.—INDIA: Maharashtra: Pune, leg. Hemant Ghate, 21.xi.2011, 18.709°N, 73.460°E, 670 m: 1 M—INDIA: Maharashtra, Pune, leg. Hemant Ghate, 18.709°N, 73.474°E, 747 m: 1 F.

*Diagnosis*. Dorsally dark brown except lateral margins of pronotum, corium and connexivum, which are completely ochraceous. Antennae black with tip of apical segment brown. Clavus and corium a little paler than body. Ocelli and first three segments of labium brown.

*Redescription*. Head 2.47 times of wider than longer, mandibular plates longer than clypeus, rounded anteriorly and meeting in front of the clypeus (Fig 9I). Eyes globular, pedunculate. Ocelli well developed. Labium four segmented, reaching middle of meso-sternum; first and third segment sub-equal, second longest and fourth smaller. Buccula prominent. Antennae five segmented, first segment cylindrical, surpassing apex of head, second and third distinctly compressed, subequal, fourth slightly dilated apically with short setae, fifth fusiform.

Pronotum dorsally finely punctate, calli less punctate, rugulose, appears smooth. Anterior margin of pronotum concave, lateral margin moderately straight, slightly raised. Humeral angles rounded. Pro-sternum sulcate in the middle, lateral area flat. Mesosternum and metasternum smooth on disk with a shallow median groove. Femora laterally flattened and spined; all tibiae sulcate with more prominent and numerous spines on both sides; all tarsi with first and third segments sub-equal, median shortest; first segment of tarsus with dense setae underneath, claws and pulvulli well developed, claws divergent.

Scutellum 0.95 times wider than long, apex rounded, with sparse punctures, deeply punctate basally with many longitudinal rugae.

Male genitalia. Pygophore slightly broad, ventral rim moderately straight (Fig 10I), ventrally medially raised (Fig 11I). Parameres highly sclerotized with rugae, outer margin globular and its tip narrowed, setose, inner margin straight (Fig 11I).

*Geographical distribution*. Cambodia, China, Indonesia, India, Laos, Malaysia, Myanmar, Philippines, Singapore, Sri Lanka, Thailand, and Vietnam. This species is reported from two extreme points of India, Nagaland (Eastern Himalayas) and Maharashtra (Western Ghats) [27, 44].

*Remarks*. This is a rediscovery of *Coridius fuscus* (Westwood, 1837) in India, 120 years after its last report by Disant (1902). *Aspongopus* (= *Coridius*) *fuscus* (Westwood, 1837) and the Indo-Burmese species *Aspongopus* (= *Coridius*) *sanguinolentus* (Westwood, 1837) are two very similar species, but the former is somewhat elongate and the latter is larger. In a recent revision of *Coridius* (Durai [27]), the distribution of *C*. *fuscus* is mentioned as Cambodia, Indonesia, Laos, Malaysia, Myanmar, and Vietnam. Some morphological differences are also noted in the redescription. According to the descriptions by Distant [44], the connexivum of *C*. *fuscus* is completely ochraceous without any bands, but according to Durai [27], the connexivum has black bands in the intersegments. All specimens collected during this study also agree with Distant [44]. However, the shape of the dissected pygophore in our collections matches well with the drawings provided by Durai [27]. This suggests that there is a possibility that *C*. *fuscus* has two color morphs. We are providing illustrations of this species for the first time, with notes on the male genitalia.

**Fig 13:**
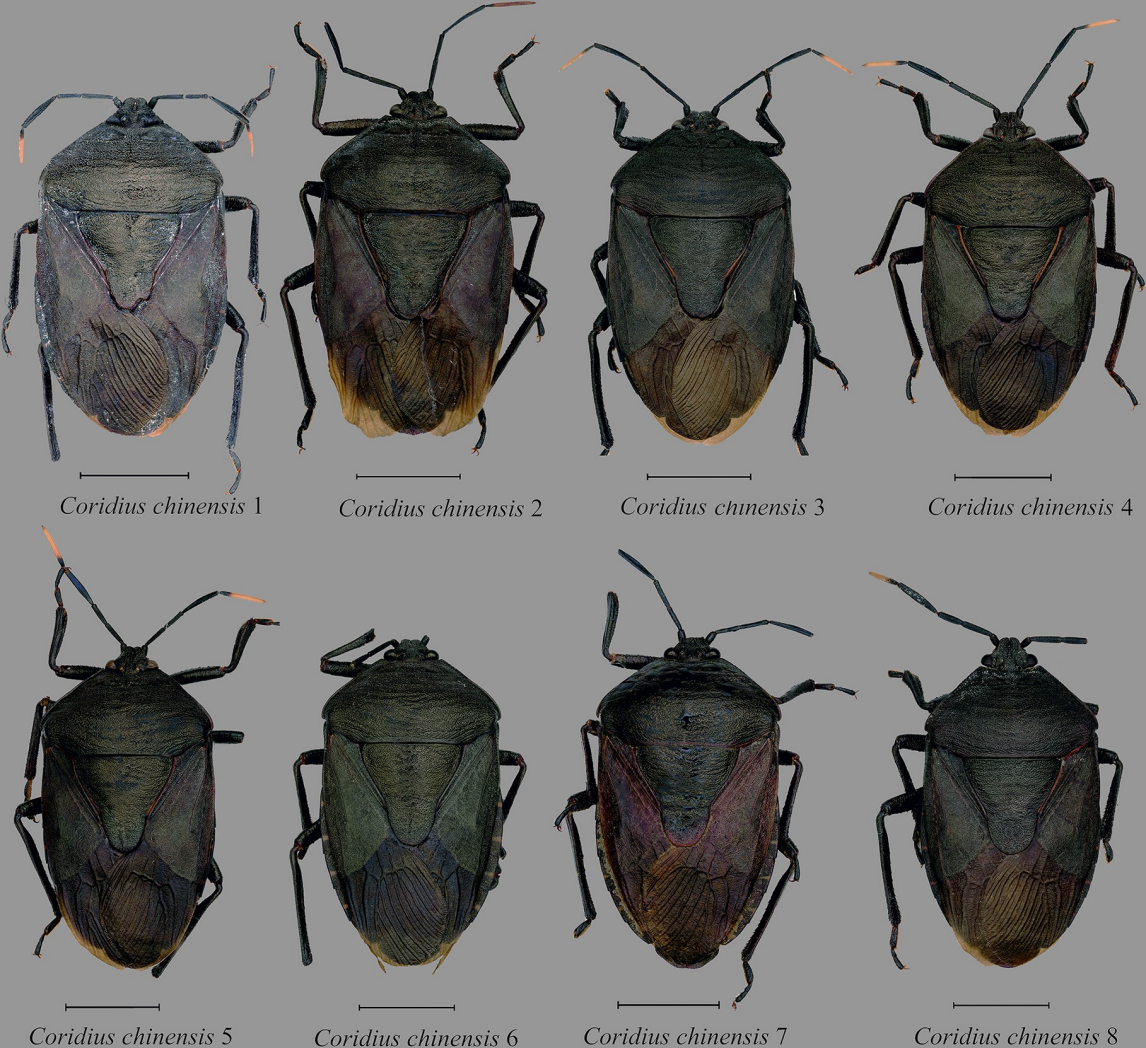
Dorsal habitus of *Coridius chinensis*. Variation within *C*. *chinensis* complex. (Scale bar = 5 mm).

**Fig 14:**
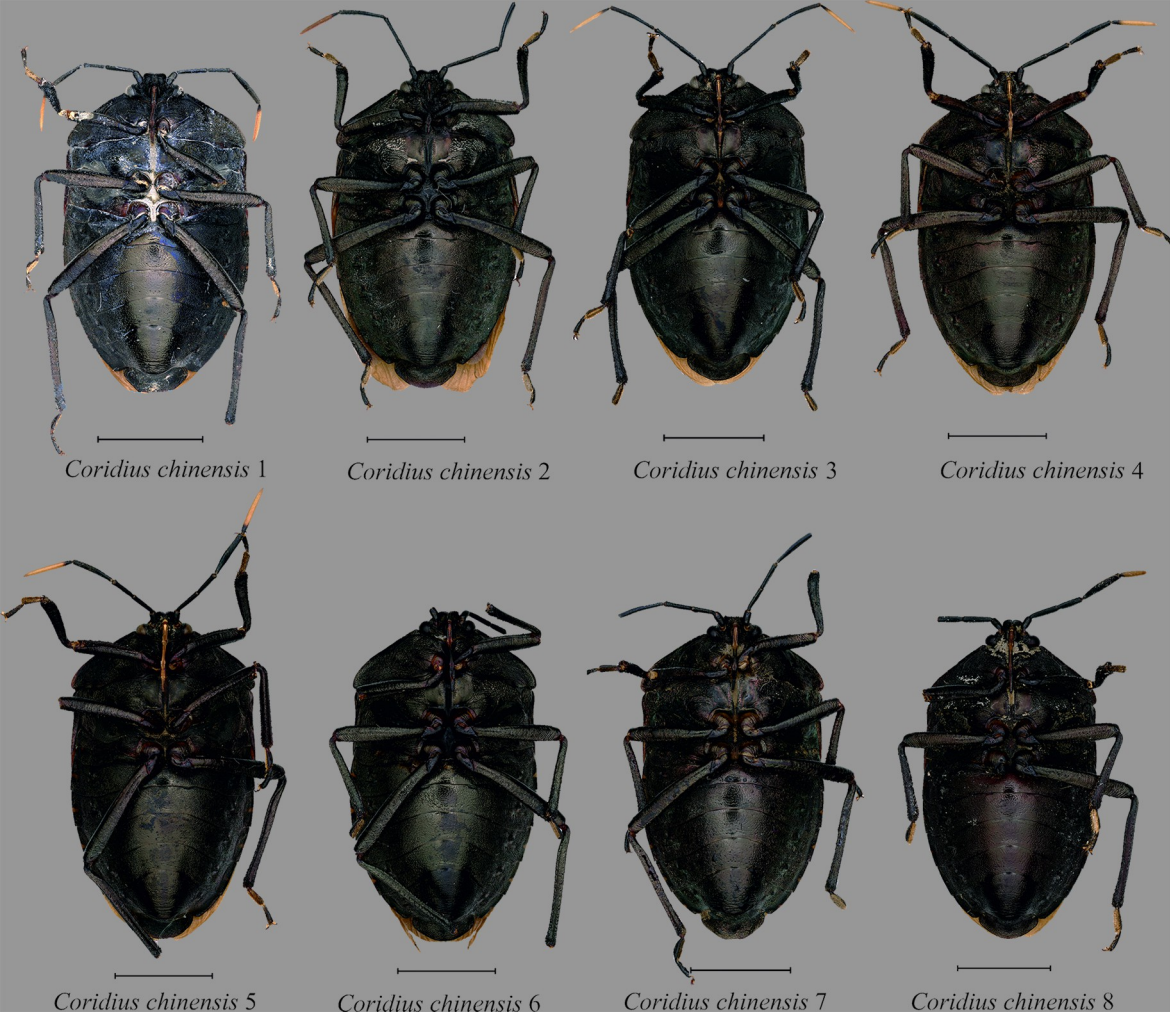
Ventral habitus of *Coridius* spp. Variation within *C*. *chinensis* complex. (Scale bar = 5 mm).

**Fig 15:**
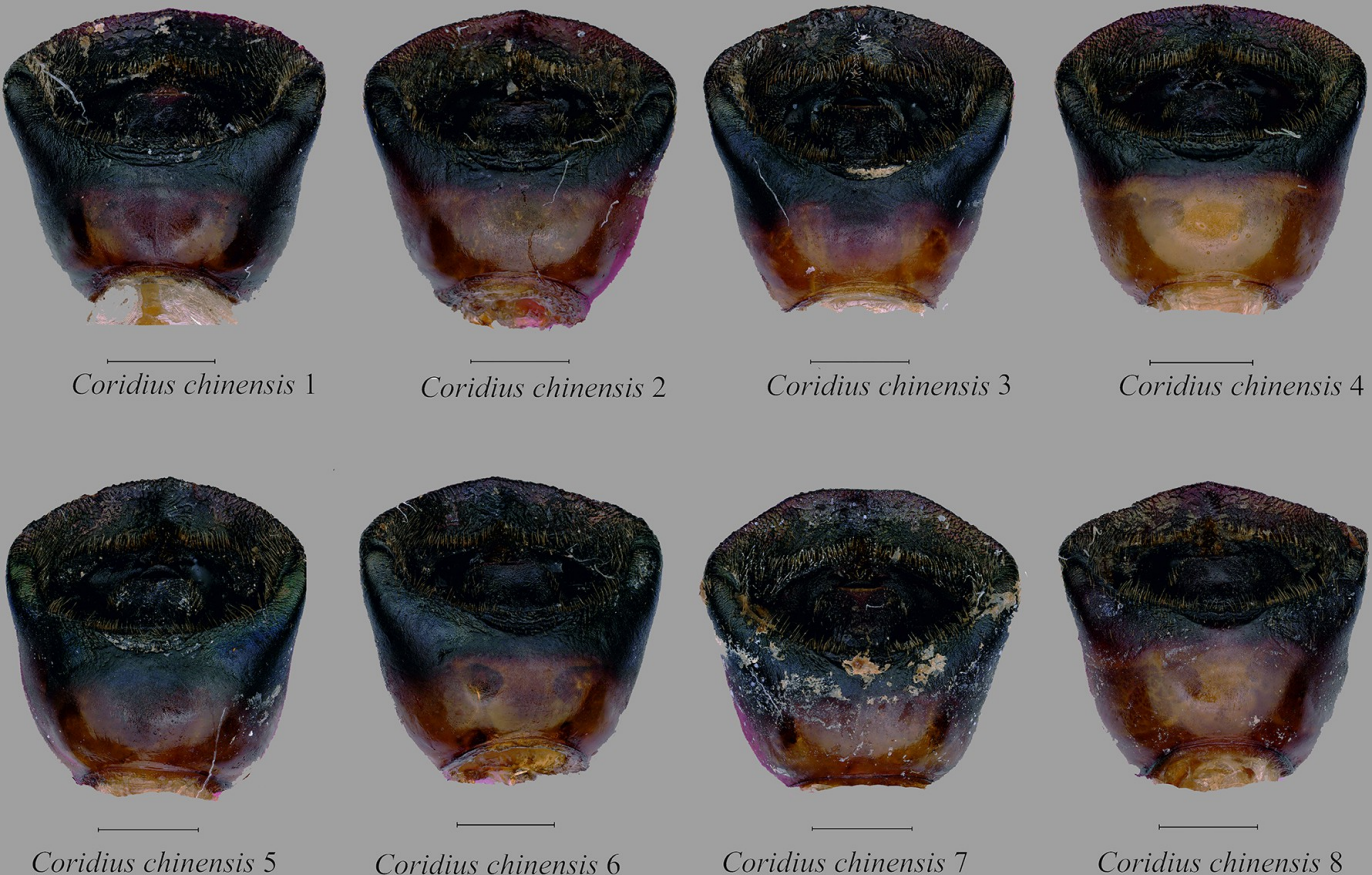
Dorsal views of male genitalia of *Coridius chinensis*. (Scale bar = 1 mm).

**Fig 16:**
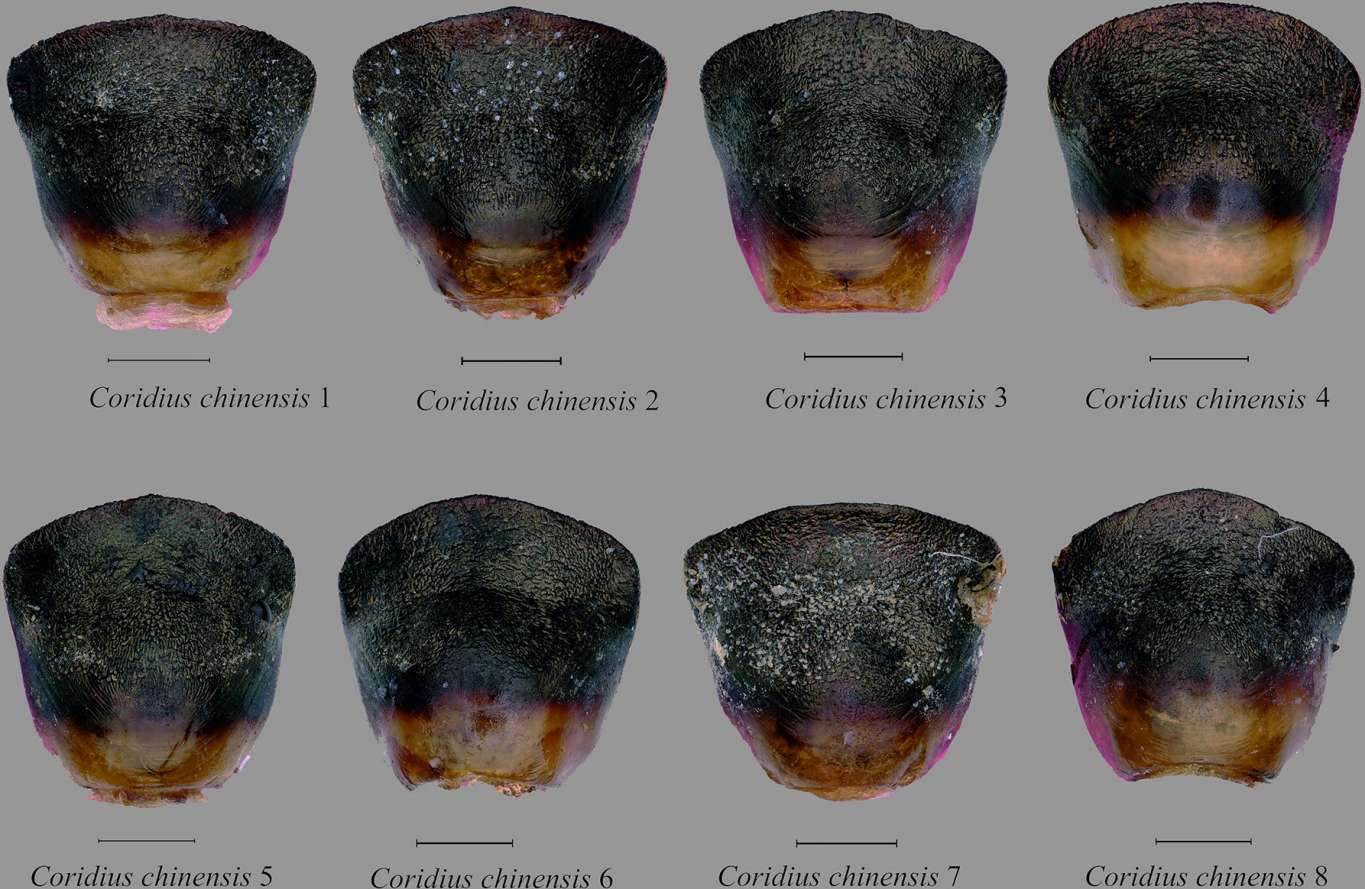
Ventral views of male genitalia of *Coridius chinensis*. (Scale bar = 1 mm).

**Fig 17:**
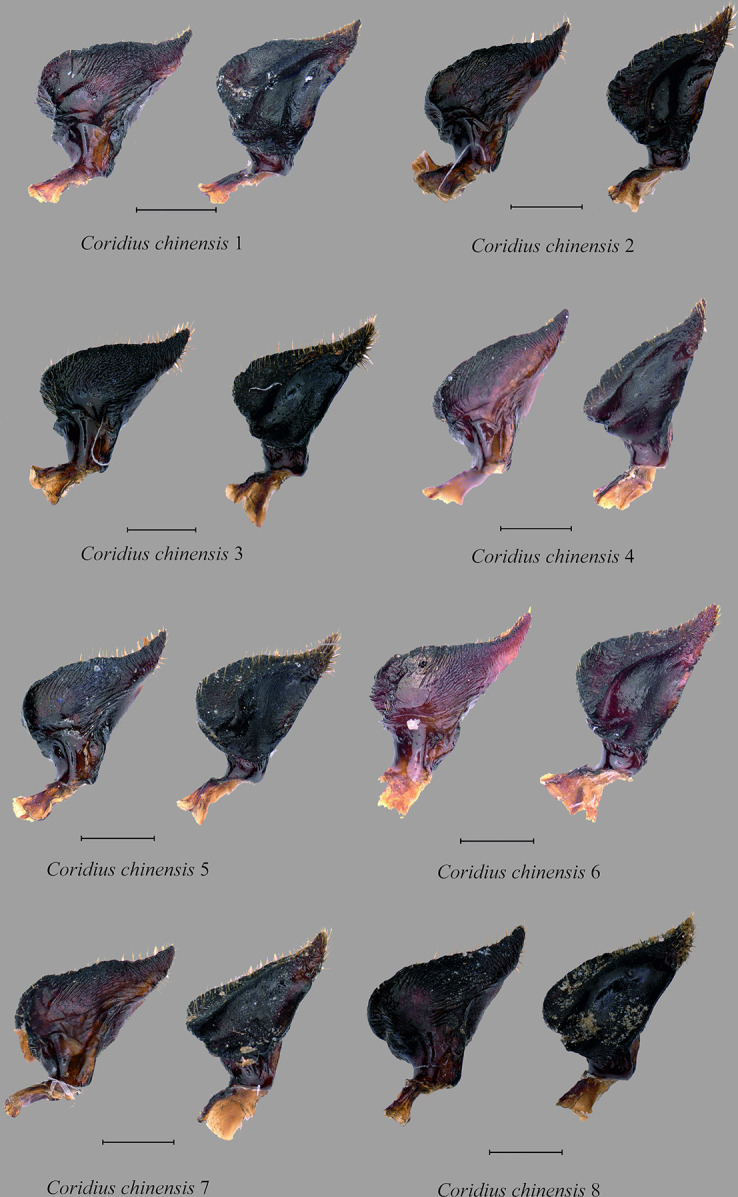
Parameres of *Coridius chinensis*. (Scale bar = 1 mm).

***Coridius chinensis* (Dallas, 1851)** (Figs 7E, 9K, 13–17).

*Aspongopus chinensis* Dallas, 1851: 349; Walker, 1868: 483; Stål, 1870: 85; Horváth, 1879: 145; Lethierry & Severin, 1893: 236; Distant, 1902: 285; 1903: 238; Oshanin, 1906: 162; Kirkaldy, 1909: 255; Oshanin, 1912: 19; Matsumura, 1913: 7; Paiva, 1919: 355; Distant, 1921: 166; Esaki, 1926: 152; Matsumura, 1930: 113, pl. 11, Fig 26; 1931: 1177; Hoffmann, 1932: 9; Strickland, 1932: 873, 874, 876; Lindberg, 1934: 14; Yang, 1934: 70–72, Fig 10; Hoffmann, 1935: 119, 176; Miller, 1956: 38; Takara, 1957: 33; Yang, 1962: 46, 47, pl. 8, Fig 67; Chang, 1974: 356; Hsiao *et al*., 1977: 70, pl. 8, Fig 123; Zhang, 1985: 53, 54, pl. 11, Fig 25; Zhang & Lin, 1988: 84, 85; Hua, 1989: 43; Zheng & Jin, 1990: 142.

*Aspongobus* [sic] *chinensis* Dohrn, 1859: 21.

*Aspongopus ochreus* Shiraki, 1910: 111, pl. 40, Fig 14.

*Aspongopus* (*Aspongopus*) *chinensis* Schouteden, 1913: 7; Tang, 1935: 355, 356.

*Cyclopelta parva* Shiraki, 1913: 207.

*Coridius chinensis* Yang, 1940: 7, 10–19, Figs 1–4; Hoffmann, 1948: 22; Stichel, 1962a: 725; 1962b: 205; Durai, 1986: 5; 1987: 190, 197, Figs 176–181, 265; Schaefer, 1987: 161; Zhang & Lin, 1987: 76; Hirashima, 1989: 183; Lis, 1990: 114, 141; 1991: 84, 87, 89, Fig 5; 1992: 37; Rolston *et al*., 1996: 35, 36, 98; Ahmad *et al*., 1997: 305, 307–310, 319 Figs 1A–1D.

Material examined: *Paralectotype Female*: CHINA: Labels: // Type // *ASPONGOPUS CHINENSIS*, 5113, des. J. Lis, PARALECTOTYPE.

INDIA: Nagaland, Viswema, leg. Aavika Dhanda, 27.iv.2019, 25.565°N, 94.147°E, 1650 m: 1 M, 1 F.—INDIA: Nagaland, Wokha, leg. Ajano Tsanglao, 20.ii.2019, 26.089°N, 94.196°E, 900 m: 1 F.—INDIA: Manipur, Chandel, leg. Barkha Subba, 6.ii.2019, 24.318°N, 94.044°E, 960 m: 1 F.—INDIA: Arunachal Pradesh: Yingkiong, leg. Gumnya Ete, 10.xii.2019, 28.632°N, 95.017°E, 275 m: 2 F.—INDIA: Manipur, Ukhrul, leg. Swapnil Boyane, 1, ii.2019, 25.095°N, 94.361°E, 775 m: 1 M, 1 F.—INDIA: Nagaland, Khonoma, leg. Ajano Tsanglao, 26.iv.2019, 25.651°N, 94.022°E, 1517 m: 1 F.

*Diagnosis*. Dorsally dark brown or black with greenish tinge (in alcohol), antennae black except fifth segment which is ochraceous, but it is one fourth base and apex black; legs dark brown or castaneous, Ventral coloration similar to dorsal side with distinct green tinge at least in artificial light (Figs 13 and 14).

*Redescription*. Head transverse, 0.41 times wider than longer, almost of same width as anterior pronotal angles; lateral margins of head in front of eyes distinctly sinuate, so that head apex looks rounded at apex. Mandibular plates longer than clypeus, meeting in front of it (Fig 9K), transversely rugulose, coarsely punctate. Clypeus transversely ridged. Eyes moderately large, pedunculate. Ocelli are prominently closer to eyes than to each other; area between eyes and ocelli coarsely punctate on the disk, slightly less coarsely punctate in front of ocelli. Antennae with scattered black setae especially on second to fourth segments, first segment slightly surpassing apex of head, cylindrical, second and third flattened with fine rugae, fourth also flattened and slightly dilated beyond middle, fifth ochraceous and densely setose with dense, short and sparse, long setae. Labium four segmented, first segment reaching base of head, fourth segment reaching about middle of mesosternum.

Pronotum moderately sloping, rhomboidal; anterior margin gently rounded behind head, anterior angles obtuse, lateral margins straight, raised as carinae; surface coarsely punctate and rugulose; calli less punctate, smooth, rugulose. Humeral angles rounded, dorsally faintly raised on corners, posterior margin straight. Posterior margin of pronotum straight.

Prosternum coarsely punctate medially, pleura finely and rugulosely punctate. Meso-sternum medially sulcate with two broad shining smooth patches on either side of midline, lateral area rugulose with scattered punctures. Metasternum narrow with a medial sulcus between meta coxae.

Scutellum rugulose punctate, 0.98 times wider than long, slightly tumescent in posterior half, apex broadly rounded.

Hemelytra. Clavus broad at base, narrowed distally and shorter than scutellum; corium broad, finely rugulose punctate. Membrane broad, with a series of basal cells and many parallel longitudinal veins. Hemelytra surpasses tip of abdomen.

Femora laterally compressed, punctate, ventrally with spinules. Tibiae sulcate with more prominent and numerous spines. Tarsi well developed, first and third segment sub-equal, middle shortest; first segment with dense mat of setae underneath, claws and pulvulli well developed; claws divergent.

Abdomen slightly wider in front of middle of its length, slightly thinner than humeral angles. Abdomen very finely punctate and rugulose, less punctate medially and appears smooth. Seventh sternum longest, posterior third rugulose, setose at posterior border, covering convex ventral portion of pygophore. Eighth sternum not visible externally in males. Spiracles prominent, situated closer to anterior border than to lateral margins of segments. Trichobothria posterior to spiracles.

Male genitalia. Exposed portion of pygophore finely rugulose, punctate ventrally with sparse long brown setae, margin finely denticulate. Anterior third of pygophore ochraceous, posterior two thirds more sclerotized, black. Ventral rim of the pygophore triangular, rounded or medially raised and straight, denticulate and without long setae (Fig 15). Ventrally moderately convex, with punctures and without setae (Fig 16). Parameres highly sclerotized with rugae, medially broad and rounded and apically narrowing, outer margin serrated and setose apically, inner margin straight (Fig 17) and with lobe basally.

*Geographical distribution*. Bhutan, China, India, Indonesia, Japan. Laos, Myanmar, Taiwan, and Vietnam. This species is widely distributed in India’s Southern (Tamil Nadu) and North-eastern regions (Assam, Manipur, Nagaland, and Arunachal Pradesh) [24, 27, 42, 44].

*Remarks*. The umami taste of *C*. *chinensis* makes it a popular dish among ethnic tribes in Northeast India. However, consuming this bug can cause problems like dizziness, nausea, and vomiting in some people. It can lead to a loss of strength and a condition of semi-consciousness in certain people (Boyane, personal communication with native people).

Different morphs in *C*. *chinensis* (Figs 13–17).

*Coridius chinensis* 1: head including ocelli, antennae except apical segment, pronotum, and scutellum brown. Hemelytra a little paler than rest of the body. Venter black, except rostrum and coxal area. Second segment of antennae (1.54 mm) longer than third (1.13 mm). Pygophore: ventral rim rounded; dorsal rim slightly concave. Parameres medially broad, tip upwardly directed with a few setae.

*Coridius chinensis* 2: head including ocelli, antennae (except apical segment), pronotum and scutellum dark black. Clavi, coria and claws brown. Membrane paler than corium. Second segment of antennae longer (1.59 mm) than third (1.39 mm). Venter completely black. Pygophore: ventral rim slightly raised medially, dorsal rim concave with setae. Parameres slightly slenderer when compared to *Coridius chinensis* 1; tip pointed with numerous setae.

*Coridius chinensis* 3: head (except ocelli), pronotum, antennae except apical segment, pronotum, scutellum, clavi, corium and legs are black. Ocelli yellow. Membrane and claws paler than rest of the body. Second antennal is smaller (1.34 mm) than the third segment (1.47 mm). Venter completely black. Scent gland area matte black. Pygophore: ventral rim raised slightly as in *Coridius chinensis* 2, dorsal rim concave. Parameres slender; tip setose and upwardly directed.

*Coridius chinensis* 4: coloration similar to *Coridius chinensis* 3. Ocelli yellow. Ventrally black except coxae and rostrum. Second antennal segment longer (1.52 mm) than third (1.21 mm). Pygophore: ventral rim rounded, dorsal rim slightly straight. Parameres slender and pointed apically.

*Coridius chinensis* 5: dorsal and ventral coloration is similar to *Coridius chinensis* 3 and *Coridius chinensis* 4. Ocelli yellow. Second antennal segment longer (1.38 mm) than third (1.26 mm). Pygophore: ventral rim raised medially, as in *Coridius chinensis* 3. Parameres medially broad and rounded; tip setose and upwardly directed.

*Coridius chinensis* 6: head including ocelli, pronotum, legs, scutellum, clavi, coriua and membrane black. Second antennal segment longer than third. Venter black except the coxal region which is brown. Pygophore like *Coridius chinensis* 2. Parameres medially broad, tip pointed and upwardly directed.

*Coridius chinensis* 7: head, antennae except apical segment, pronotum, scutellum and legs black. Clavi, coria, membrane, and tip of scutellum brown. Venter black. Pygophore: ventral rim medially weakly elevated and straight, dorsal rim concave. Parameres outer margin rounded, tip upwardly directed.

*Coridius chinensis* 8: head, antennae (except apical segment), pronotum, scutellum, clavui, coriua and legs black. Membrane paler than color of the body. Rostrum, coxae, prosternum, and abdomen segments II-VI brown. Second antennal segment longer than third. Pygophore: ventral rim medially raised, triangular-like, dorsal rim almost straight. Parameres with pointed as in morphs of *C*. *chinensis*.

Remarks: Eight samples of *Coridius chinensis* (Dallas, 1851) formed six lineages in our analysis. Although all samples are keyed out to *Coridius chinensis*, they show slight variations in the body color and structure of pygophores and parameres. We studied the paralectotype of *C*. *chinensis* and found that the basal ¼ of the fifth antennal segment is black in all specimens. *C*. *chinensis* 3–5 have yellow ocelli, while all other have black ocelli. These three samples, which share similar morphology, were collected from the same state.

### Key to the species of *Coridius* (modified from Durai (1987); Ahmad *et al*., (1997))

1. Antennae 4 segmented; body length more than 24 mm, body cupreous; 2^nd^ antennal segment longest (Fig 5A); pygophore wider than long (Fig 10A); parameres medially stout, apex tapering with long seta (Fig 12A)………………….*C*. *insperatus* **sp. nov.**

- Antennae 5 segmented; body length less than 24 mm, body colour variable; 4th antennal segment longest; shape of pygophore and paramere different………………………………………………………………………….…… 2

2. Second antennal segment shorter than third; body length more than 20 mm; entire body reddish brown; lateral margin of pronotum rounded (Fig 5D); ventral rim of pygophore medially weakly elevated (Fig 10D); outer margin of paramere strongly rounded, apex narrowed, setose (Fig 12D) ….………………………….*C*. *nepalensis*

- Second antennal segment longer than third; body length 16–20 mm; body dark brown, blackish brown, ochraceous or black; lateral margin of pronotum straight or moderately curved; ventral rim of pygophore emarginate or convex; outer margin of parameres moderately rounded, apex variable ………….……………………………3

3. Body length 18–20 mm; mandibular plates not meeting in front in clypeus; entirely blackish brown (Fig 5C); pygophore slightly medially elevated (Fig 10C); parameres short, apex blunt (Fig 12C) ……………… .… .… .… .… .*C*. *esculentus* **sp. nov.**

- Body length less than 18 mm; mandibular plates meeting in front of clypeus; colour variable; pygophore and parameres variable……………….………………… 4

4. Basal fourth of fifth antennal segment black (Fig 13); outer margin of paramere medially broad and basally with knob like structure, apex thumb-like upwardly directed (Fig 17) …… .… .… .… .… .… .… .… .… .… .… .… .… .… .… .… .… .… .… .… .……*C*. *chinensis*

- Basal fourth of fifth antennal segment not black; outer margin of paramere variable and basally without knob like structure, apex not thumb-like or upwardly directed………………………………………………………………………………. 5

5. Antennae entirely black or of the same color as head………….………… .… .. 6

- Antennae with orange, yellowish brown or ochraceous segment five……… 10

6. Lateral margins of pronotum and connexivum black; body uniformly black; pronotum trapezoidal (Fig 5G).…………………………………………. *C*. *assamensis*

- Lateral margins of pronotum and connexivum red, orange, or yellow; body colour variable; pronotum shape variable …………………………………………. 7

7. Pronotum, apical half of scutellum, and coria yellowish orange (Fig 5H); abdomen ventrally reddish (Fig 6H); body more than 17 mm …………………*C*. *ianus*

- Pronotum, apical half of scutellum, and coria black or blackish brown; abdomen ventrally black; body less than 17 mm ………………….…………….……8

8. Connexivum, and posterior third of pygophore, orange (Fig 7A); fourth segment of antennae flat and dilated.……………………………………… *C*. *laosanus*

- Connexivum, and posterior third of pygophore, reddish or black; fourth segment of antennae slender and two apical thirds dilated ………………….……… 9

9. Pronotum dark brown with red band along lateral margin; connexivum bright red; abdomen ventrally dark brown. (Fig 7B) ……………………… *C*. *sanguinolentus*

- Pronotum black with ochraceous band along lateral margin; connexivum ochraceous; abdomen ventrally black. (Fig 5I) …………….………………. C. fuscus

10. Scutellum wider than longer…………………………….……….……….… 11

- Scutellum longer than wider…………………………………………………. 12

11 Body uniformly black and coria brown; second antennal segment shorter than third (Fig 7C)… .… .… .… .… .… .… .… .… .… .… .… .… .… .… .… .… .… .… .… .… .…… *C*. *nigriventris*

- Body ochraceous or blackish brown and coria of the same color as pronotum; second antennal segment longer than third (Fig 5F) ……………………. C. brunneus

12. Pronotum with mid-longitudinal pale ochraceous band; antennal segments 2 to 4 indistinctly grooved; body pale brown to dark brown with irregular patches of yellow (Fig 5B); pygophore slightly narrow and distally wide, ventral rim slightly notched medially (Fig 10B); outer margin of parameres moderately rounded, apex narrowed and upwardly directed (Fig 12B)………… .… .… .… .… .… .… .… .… .… .… .… .*C*. *adii* **sp. nov.**

- Pronotum without mid-longitudinal band; antennal segments 2 to 4 distinctly grooved; body yellowish brown with lateral margin of head blackish brown (Fig 5E); pygophore proximally narrow and distally wide, ventral rim not notched medially (Fig 10E); outer margin of parameres strongly rounded and apex robust and straight (Fig 12E)…………………………………………………………….………C. singhalanus

## Discussion

### Phylogenetic consideration

In our analyses, we observed robust family-level relationships within the Pentatomoidea superfamily. Specifically, we found that Dinidoridae formed a monophyletic group and sister taxa to Tessaratomidae. The previous studies showed Dinidoridae does not form a sister relationship with Tessaratomidae [45], whereas our results, as well as a few studies [46, 47], supported the sister relationship between these taxa.

In the integrated framework, our findings from molecular and morphological studies revealed a high convergence in the species delimitation of most *Coridius* species. Tree-based delimitation PTP and bPTP retrieved 13 and 14 lineages, respectively, including three new species. Except for *C*. *chinensis*, which formed six putative species. Similarly, ASAP 1 recovered 14 and ASAP 2 recovered 25, respectively. In *C*. *adii* **sp. nov.** *C*. *assamensis*, *C*. *chinensis*, and *C*. *singhalanus*, we found discrepancies between morphological and molecular delimitation analyses. Therefore, we compared the parameres of these species, which showed distinctness; however, species delimitation PTP, bPTP, and ASAP 1 indicated a single species, whereas ASAP 2 treated them as distinct species (Fig 4).

Similarly, we observed that *C*. *esculentus* **sp. nov.** forms a clade with *C*. *chinensis* and is closely related to it in terms of coloration. However, PTP and bPTP grouped this clade as a single species, while ASAP 1 and ASAP 2 treated *C*. *esculentus* **sp. nov.** and *C*. *chinensis* as separate species (Fig 4). Additionally, a closer look at the head and pygophore revealed that the major difference between *C*. *esculentus* **sp. nov.** and other species is in the shape of the mandibular plates and the parameres (Fig 12). It’s important to note that the low node support for *C*. *esculentus* **sp. nov.** (PP: 0.82, MLBS: 52) is likely a result of the small sample size. A well-supported clade of *C*. *nepalensis* and two unidentified species resulted in a single species in the bPTP analysis. We could not examine the voucher specimens for unidentified species sequences, which create gaps in Fig 4. In the instance of *C*. *ianus*, all our analyses showed two putative species. *C*. *ianus* has several colour and size variants; it would be interesting to sequence multiple specimens to understand their relationships, but this remains out of the scope of the present study. The African species *C*. *viduatus*, which morphologically resembles *C*. *ianus*, formed a sister clade with each other (Fig 4). Our results also reveal that *C*. *nigriventris*, which was previously synonymized with *C*. *nepalensis*, forms a distinct lineage, and therefore we suggest treating that as a valid species.

### Hidden diversity in *Coridius chinensis*

The current study shows that *C*. *chinensis* is a complex of species and, at the same time, disentangles some of its morphological diversity within north-eastern India. We found morphological variation along the geographical distribution of specimens of the *C*. *chinensis* complex and discovered at least six independent lineages, which were earlier grouped as one valid species, *C*. *chinensis*. The construction of trees using maximum likelihood and Bayesian inference for the several specimens of *Coridius chinensis* revealed similar tree topologies, with each clade being well-supported by high posterior probability and bootstrap support (see Fig 4). The phylogenetic placement of *C*. *chinensis* individuals may suggest that they are partially and geographically differentiated lineages of a species.

Our findings for *C*. *chinensis* indicate that there is high intraspecific diversity resulting in the six putative species. These individuals are from diverse geographic areas and may have different ecological niches and food preferences. Given this, we observed that *C*. *chinensis* is genetically diverse yet morphologically indistinguishable (Figs 13–17). Since the morphological investigation did not reveal any significant differences, we opted to maintain its current taxonomic status. To address the complexity within *C*. *chinensis*, future research will require larger taxon sampling across the occurrence regions, as well as multiple loci (nuclear and mitochondrial) data combined with ecological data. We agree that the use of additional nuclear gene markers can provide a well-resolved tree and gain a much deeper understanding of the phylogenetic relationships.

### Taxonomic consideration

Durai [27] catalogued 37 species under the genus *Coridius* and listed all synonyms and distribution records for each species. Later, Lis [23] published a checklist of the Old World Dinidoridae species, including *Coridius*, along with a few new synonyms, and proposed two new genera: the first was *Coridiellus* Lis 1990, to which six species of *Coridius* were transferred, and the second was *Colporidius* Lis, where *Coridius aeneus* (Walker, 1868) was transferred. Considering all these changes, Rolston *et*. *al*. [42] published a catalogue of the world’s Dinidoridae, which included only 32 valid species under the genus *Coridius*.

*C*. *adii* **sp. nov.** *C*. *esculentus* **sp. nov.** and *C*. *insperatus* **sp. nov.** described here, unquestionably belong to the genus *Coridius* since they exhibit all the key characters of Dinidorini and *Coridius*. Interestingly, according to Distant’s [44] and Durai’s [27] revisions, the genus *Coridius* has five segmented antennae. But *C*. *insperatus* **sp. nov.** described from Arunachal Pradesh, has only four segmented antennae that distinguish it from all other known species. The mandibular plates in *C*. *insperatus* **sp. nov.** are narrower and mesially meeting each other (Fig 9A), whereas in all other species they are broad and meet in front of the clypeus (Fig 9B–9K).

The Indian *Coridius* can be divided into two groups solely based on antennal coloration (though this has not been tested phylogenetically): the species with all antennal segments black form the first group, which includes *C*. *assamensis*, *C*. *fuscus*, *C*. *ianus*, *C*. *laosanus*, *C*. *nigriventris*, and *C*. *sanguinolentus*. In the second group, the apical segment of the antenna is orange or yellowish brown. This includes *C*. *adii* **sp. nov.,** *C*. *chinensis*, *C*. *brunneus*, *C*. *esculentus* **sp. nov.,** *C*. *insperatus* **sp. nov.,** *C*. *nepalensis*, and *C*. *singhalanus*.

With its orange apical antennomeres, *C*. *adii* **sp. nov.** and *C*. *esculentus* **sp. nov.** fall into the second group. Though *C*. *adii* **sp. nov.** seems unrelated to the group with entirely black antennae, it resembles *C*. *singhalanus* in body coloration but differs in anatomy. The pygophores of *C*. *adii* **sp. nov.** and *C*. *singhalanus* are almost equal in length and width, but the ventral rim is distinctly notched (Fig 10B) in *C*. *adii* **sp. nov.** while it is not notched in *C*. *singhalanus* (Fig 10E). Similarly, the parameres in *C*. *adii* **sp. nov.** have a broadly rounded outer margin and a distally tapering tip (Fig 12B), whereas in *C*. *singhalanus* it is medially broad and has the apical tip rounded (Fig 12E). Additionally, we compared *C*. *adii* **sp. nov.** with images of the lectotype of *C*. *singhalanus* (Fig 7D), which clearly showed the absence of a pale carina on the pronotum of *C*. *singhalanus*.

Similarly, *C*. *esculentus*, **sp. nov.** is included in the second group as well and has yellow apical segments; it bears a resemblance to *C*. *chinensis* due to its dark brown color. Therefore, we compared this species with the images of the *C*. *chinensis* paralectotype (Fig 7E). Upon close examination of both *C*. *chinensis* (Figs 13−17) and its paralectotype (Fig 7E), we observed a consistent pattern–a black region on the basal fourth of the apical antennal segment (Figs 7E, 7H and 13). However, in the case of *C*. *esculentus* **sp. nov.** this black region is absent (Fig 7G). Additionally, another distinguishing trait that sets *C*. *esculentus* **sp. nov.** apart from is the knob-like structure on the paramere (Fig 12C). In 1997, Ahmad *et*. *al*. [48] described two new species of *Coridius* from Pakistan, *C*. *turbatensis* Ahmad, Hussain & Kamaluddin, 1997, and *C*. *neobrunneus* Ahmad, Hussain & Kamaluddin, 1997, with which we have compared the new species; however, these species are more closely related to *C*. *ianus* and *C*. *brunneus* (see [48]).

Distant [44] included 10 species under the genus *Aspongopus* (= *Coridius*), but later Durai [27] synonymized a few species, including *C*. *nigriventris* under *C*. *nepalensis*, based on the morphological similarity of aedeagus. In our analysis, we found that these two species formed distinct clades (Fig 4); hence, we reinstated and re-established the species status of *C*. *nigriventris* based on morphological and molecular evidence (Figs 7 and 8C) (for more information, refer to the results section). The most useful diagnostic characters for distinguishing species were revealed: these are relative size, length of antennal segments, shape of mandibular plates, pygophore, and parameres.

Finally, the discovery of *C*. *adii* **sp. nov.,** *C*. *esculentus* **sp. nov.,** and *C*. *insperatus* **sp. nov.** implies that there are likely many more species to be described from the north-eastern area of India. In addition, extensive systematic surveys throughout the range of *C*. *chinensis* are required to resolve the species’ hidden diversity.

## Conclusions

There are 13 *Coridius* species known from India, including three new species described in this study. The phylogenetic trees and species delimitation results support the establishment of three new *Coridius* species. The LDA revealed that the most important morphological characters to consider while identifying these species are total body length, maximum body width, and the length and width of the pronotum and scutellum. Our findings indicate that *C*. *chinensis* is a species complex that needs extensive research to resolve intraspecific variation. In this work, we reinstated *C*. *nigriventris*, which was previously synonymized under *C*. *nepalensis*. Also, we are providing illustrations of *C*. *assamensis*, *C*. *fuscus*, *C*. *laosanus*, *C*. *sanguinolentus*, and *C*. *singhalanus* for the first time. The current study on *Coridius* is intended to provide a framework for future studies with a larger sample size as well as to demonstrate that our integrative approach combining morphological (LDA) and molecular analyses (species delimitation methods) is most promising for resolving species delimitation and uncovering hidden diversity. The presence of several undescribed lineages in our data highlights the need to revise the taxonomy and systematics of *Coridius*. A large-scale global phylogenetic investigation of all known *Coridius* likely has great potential to yield numerous additional species.

## Supporting information

S1 File Measurements morphological characters of *Coridius* spp. (DOCX)

S1 Fig Variation in morphometric characters of *Coridius* spp. (A) Pronotum length and breadth ratio (PL/PB). (B) scutellum length and breadth ratio (SL/SB). (C) maximum body breadth (MB). (D) total body length (TBL).
(TIF)

S2 Fig Correlation plots of different variables. (TIF)
