# Supplementary material for: Integrative taxonomy of the genus Coridius Illiger, 1807 (Hemiptera: Heteroptera: Dinidoridae) reveals hidden diversity and three new species from North-East India
Source: PLoS One. 2024 Jul 31;19(7):e0298176. doi: 10.1371/journal.pone.0298176 (PMC11290622; doi:10.1371/journal.pone.0298176)
Supplement: S1 File — (DOCX) [file pone.0298176.s001.docx]

**Integrative taxonomy of the genus *Coridius* Illiger, (Hemiptera: Heteroptera: Dinidoridae) reveals hidden diversity and three new species from North-East India**

Swapnil S. Boyane^1,2^, Sandeep Sen^3^, Dharma Rajan Priyadarsanan^1*^, Pavan Kumar Thunga^1^, Nikhil U. Joshi^1^ and Hemant V. Ghate^4^

^1^*Ashoka Trust for Research in Ecology and the Environment (ATREE), Royal Enclave, Srirampura, Jakkur Post, Bangalore 560064, India*.

Present address: ^2^*Department of Biological Sciences, Texas Tech University, Lubbock, 79409, Texas, USA.*

^3^*Mountain Science Center, University of Tsukuba, 1278-294 Sugadairakogen, Ueda, Nagano 386-2204, Japan.*

^4^*Post Graduate Research Centre, Department of Zoology, Modern College of Arts Commerce and Science, Shivajinagar, Pune 411005, Maharashtra, India.*

**Supplementary file 1**

**Measurements are given in the millimeter (mm).**

***Coridius*** ***insperatus,* Boyane, Ghate & Priyadarsanan, sp. nov.**

Measurements (in mm) – Total length 23.5; head length 1.75, head width 3.69, dorso-median length of pronotum 5.9; breadth of pronotum 12; antenna: length of first segment 1, second segment 3.89, third segment 2.35, fourth segment 2.51; labium: total length 4.75, medial length of scutellum 7.01, basal width of scutellum, 6.97; Fore leg: femur 4.54, tibia 4.55, tarsus with claws 2.80; mid legs: femur 5.52, tibia 5.30, tarsus with claws 2.27; hind leg femur 7.18, tibia 7.58, tarsus with claws 3.28; length of hemelytra 17.69; maximum width of abdomen 12.97; medial length of Pygophore 3.78, maximum width of pygophore 4.15; Paramere length - 1.48mm,median width paramere- 0.75.

***Coridius*** ***adii,* Boyane, Ghate & Priyadarsanan, sp. nov.**

Measurements (in mm) – Total length 17.0; head length 1.35, head width 3.0, dorso-median length of pronotum 4.0; breadth of pronotum 9.3; antenna: length of first segment 0.87, second segment 1.59, third segment 1.64, fourth segment 2.73; labium: total length 3.61, medial length of scutellum 5.41, basal width of scutellum, 5.18; Fore leg : femur 4.37, tibia 4.04, tarsus with claws 2.30; mid legs : femur 4.84, tibia 4.01, tarsus with claws 2.50; hind leg: femur 6.54, tibia 6.09, tarsus with claws- 2.58; length of hemelytra 13.76; maximum width of abdomen 9.92; Paramere length- 1.46, median width- 0.70.

***Coridius esculentus,* Boyane, Ghate & Priyadarsanan, sp. nov.**

Measurements (in mm) – Total length 18.61; head length 2.59, head width 3.24, dorso-median length of pronotum 4.38; breadth of pronotum 9.99; antenna: length of first segment 0.80, second segment 1.60, third segment 1.53, fourth segment 2.71, fifth segment 2.70; labium: total length 4.35, medial length of scutellum 5.94, basal width of scutellum, 5.46; fore legs : femur 4.50, tibia 3.90, tarsus with claws 2.30; mid legs : femur 4.74, tibia 4.36, tarsus with claws 2.29; hind legs: femur 5.74, tibia 6.16, tarsus with claws 2.56; length of hemelytra 14.91; maximum width of abdomen 10.72; medial length of pygophore 3.31, maximum width of pygophore 3.56; paramere length 1.33, median width paramere 0.79.

***Coridius nepalensis* (Westwood, 1837)**

Measurements (in mm) – Total length 21.04; head length 1.52, width 3.38; dorso-median length of pronotum 4.85, breadth 10.96; antenna: length of first segment 0.90, second segment 1.30, third segment 1.83, fourth segment 2.86, fifth segment 2.85; labium: total length 4.20; medial length of scutellum 6.38, basal width 6.38. Foreleg: femur 4.15, tibia 4.0, tarsus with claws 2.74; mid legs: femur 4.97, tibia 4.64, tarsus 2.50; hind leg: femur 6.89, tibia 6.65, tarsus with claws 2.76; length of hemelytra 16.52; maximum width of abdomen 11.97; pygophore length 3.40, maximum width 4.0; paramere length- 1.40, maximum width - 0.80.

***Coridius singhalanus* (Distant, 1900)**

Measurements (in mm) – Total length 16.88; head length 1.19, head width 2.84, dorso-median length of pronotum 4.03; breadth of pronotum 9.05; antenna: length of first segment 0.70, second segment 1.43, third segment 1.38, fourth segment 2.03, fifth segment 2.33; labium: total length 3.21, medial length of scutellum 5.25, basal breadth of scutellum, 5.12; Foreleg : femur 4.03, tibia 3.17, tarsus with claws 2.13; mid legs : femur 4.57, tibia 3.85, tarsus 2.26; hind leg): femur 5.58, tibia 5.42, tarsus with claws 2.26; length of hemelytra 13.32; maximum width of abdomen 9.82; Pygophore length 3.02, maximum width of pygophore 3.25.

***Coridius brunneus* (Thunberg, 1783)**

Measurements (in mm) – Total length 19.28; head length 1.30, head width 2.52, dorso-median length of pronotum 4.53; breadth of pronotum 9.48; antenna: length of first segment 0.70, second segment 1.21, third segment 1.23, fourth segment 2.03, fifth segment 2.05; labium: total length 2.78, medial length of scutellum 5.39, basal width of scutellum, 5.86; Foreleg : femur 3.87, tibia 3.64, tarsus with claws 2.20; mid legs : femur 4.95, tibia 4.67, tarsus 2.22; hind leg : femur 6.43, tibia 6.04, tarsus with claws 2.30; length of hemelytra 13.97; maximum width of abdomen 10.53; Pygophore length 2.07, maximum width of pygophore 3.03.

***Coridius assamensis* (Distant, 1902)**

Measurements (in mm) – Total length 16.46; head length 1.47, width 2.74; dorso-median length of pronotum 4.08; breadth 8.53; antenna length of first segment 0.70, second segment 1.50, third segment 1.05, fourth segment 2.24, fifth segment 1.63; labium: total length 3.41; scutellum : medial length 4.67, basal width 4.66; Foreleg : femur 3.39, tibia 2.83, tarsus with claws 2.21; mid legs : femur 3.89, tibia 3.39, tarsus 1.12; hind leg : femur 5.06, tibia 4.71, length of hemelytra 12.72; maximum width of abdomen 8.89; pygophore length 2.86, maximum width 3.03; paramere length - 1.13, maximum width 0.71.

***Coridius ianus* (Fabricius, 1775)**

Measurements (in mm) – Total length 17.36; head length 1.30, head width 2.52, dorso-median length of pronotum 4.53; breadth of pronotum 9.48; antenna: length of first segment 0.70, second segment 1.21, third segment 1.23, fourth segment 2.03, fifth segment 2.05; labium: total length 2.78, medial length of scutellum 5.39, basal width of scutellum, 5.86; Foreleg : femur 3.87, tibia 3.64, tarsus with claws 2.20; mid legs : femur 4.95, tibia 4.67, tarsus 2.22; hind leg : femur 6.43, tibia 6.04, tarsus with claws 2.30; length of hemelytra 13.97; maximum width of abdomen 10.53; Pygophore length 2.07, maximum width of pygophore 3.03.

***Coridius fuscus* (Westwood, 1837)**

Measurements (in mm) – Total length 17.00; head length 1.16, head width 2.87, dorso-median length of pronotum 4.01; breadth of pronotum 9.15; antenna: length of first segment 0.78, second segment 1.57, third segment 1.34, fourth segment 2.38, fifth segment 2.14; labium: total length 3.80, medial length of scutellum 5.11, basal width of scutellum, 5.43 ; Foreleg : femur 3.70, tibia 3.80, tarsus with claws 2.20; mid legs : femur 4.02, tibia 4.13, tarsus with claw 2.13; hind leg : femur 5.35, tibia 6.04, tarsus with claws 2.10; length of hemelytra 13.18; maximum width of abdomen 10; Pygophore length 2.51, maximum width of pygophore 3.21.

***Coridius sanguinolentus* (Westwood, 1837)**

Measurements (in mm) - Total length 16.87; head length 3.0, dorso-median length of pronotum 4.16; breadth of pronotum 9.36; antennal segments damaged; labium: total length 3.22, medial length of scutellum 5.16, basal width of scutellum, 5.52. Mid legs: femur 4.38, tibia 4.23; hind leg: femur 6.34, tibia 5.85; length of hemelytra 13.42; maximum width of abdomen 10.29.

***Coridius* *nigriventris* (Westwood, 1837)**

Measurements (in mm) – Total length 17.70; head length 2.86, dorso-median length of pronotum 4.35; breadth of pronotum 9.51; antenna: length of first segment 0.94, second segment 1.46, third segment 1.24, fourth segment 2.51, fifth segment 2.40; labium: total length 3.48, length of scutellum 5.16, breadth of scutellum 5.52, Mid legs: femur 5.54, tibia 4.15; hind leg : femur 6.36, tibia 5.53, length of hemelytra 14.12.

***Coridius chinensis* (Dallas, 1851)**

Measurements (in mm) – Total length 16.17; head length 1.17, head width 2.85, dorso-median length of pronotum 4.06; breadth of pronotum 9.23; antenna: length of first segment 0.60, second segment 1.50, third segment 1.04, fourth segment 2.27, fifth segment 1.85; labium: total length 3.46, medial length of scutellum 5.09, basal breadth of scutellum, 5.15; Foreleg : femur 3.34, tibia 3.38, tarsus with claws 2.40; mid legs : femur 4.47, tibia 4.16, tarsus 2.49; hind leg : femur 5.61, tibia 6.0, tarsus with claws 2.46; length of hemelytra 12.82; maximum width of abdomen 9.62.
