# Supplementary figures and images for: Integrative taxonomy of the genus Coridius Illiger, 1807 (Hemiptera: Heteroptera: Dinidoridae) reveals hidden diversity and three new species from North-East India
Source: PLoS One. 2024 Jul 31;19(7):e0298176. doi: 10.1371/journal.pone.0298176 (PMC11290622; doi:10.1371/journal.pone.0298176)

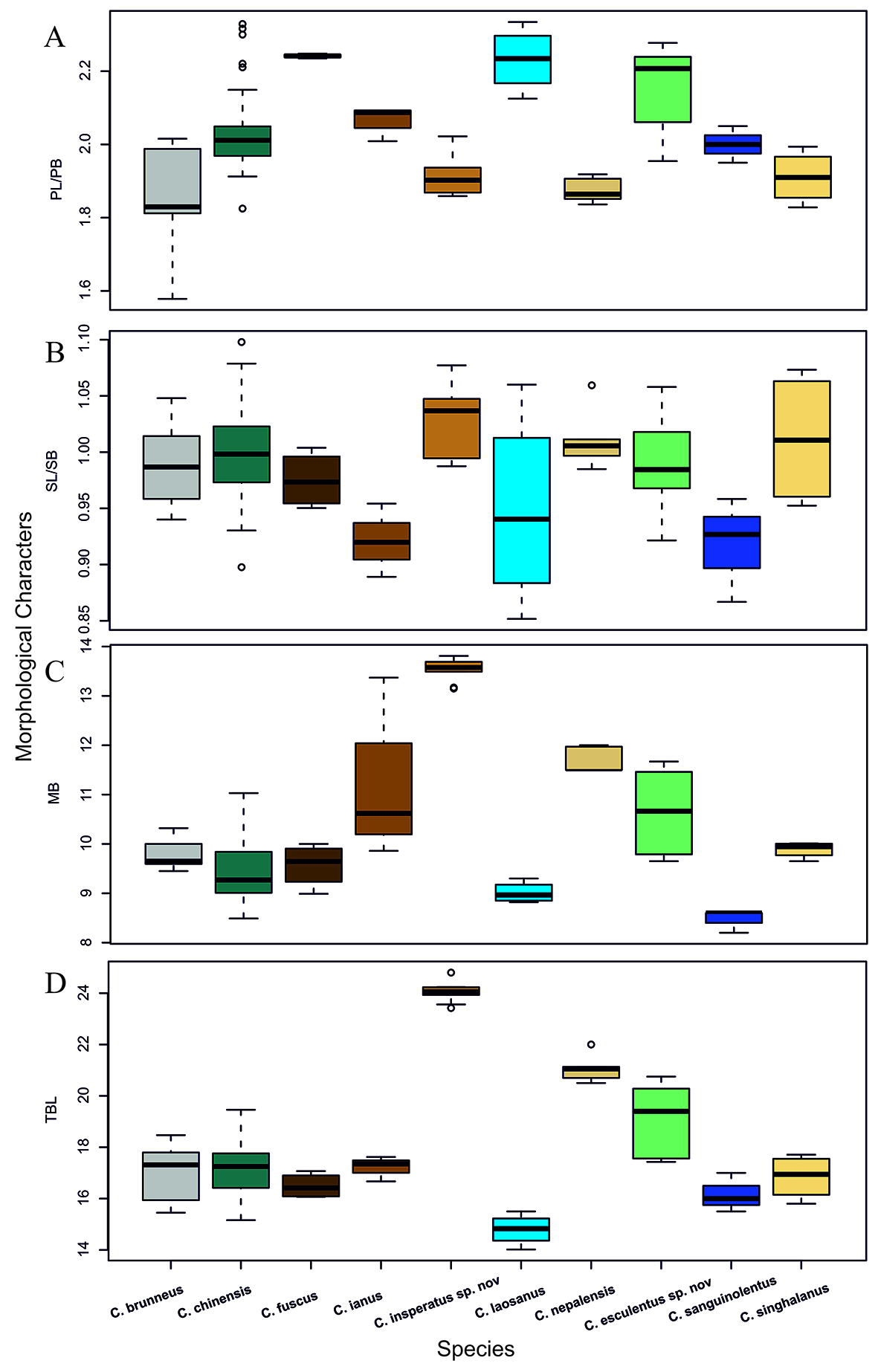

Supplement: S1 Fig — (A) Pronotum length and breadth ratio (PL/PB). (B) scutellum length and breadth ratio (SL/SB). (C) maximum body breadth (MB). (D) total body length (TBL). (TIF) [file pone.0298176.s002.tif]

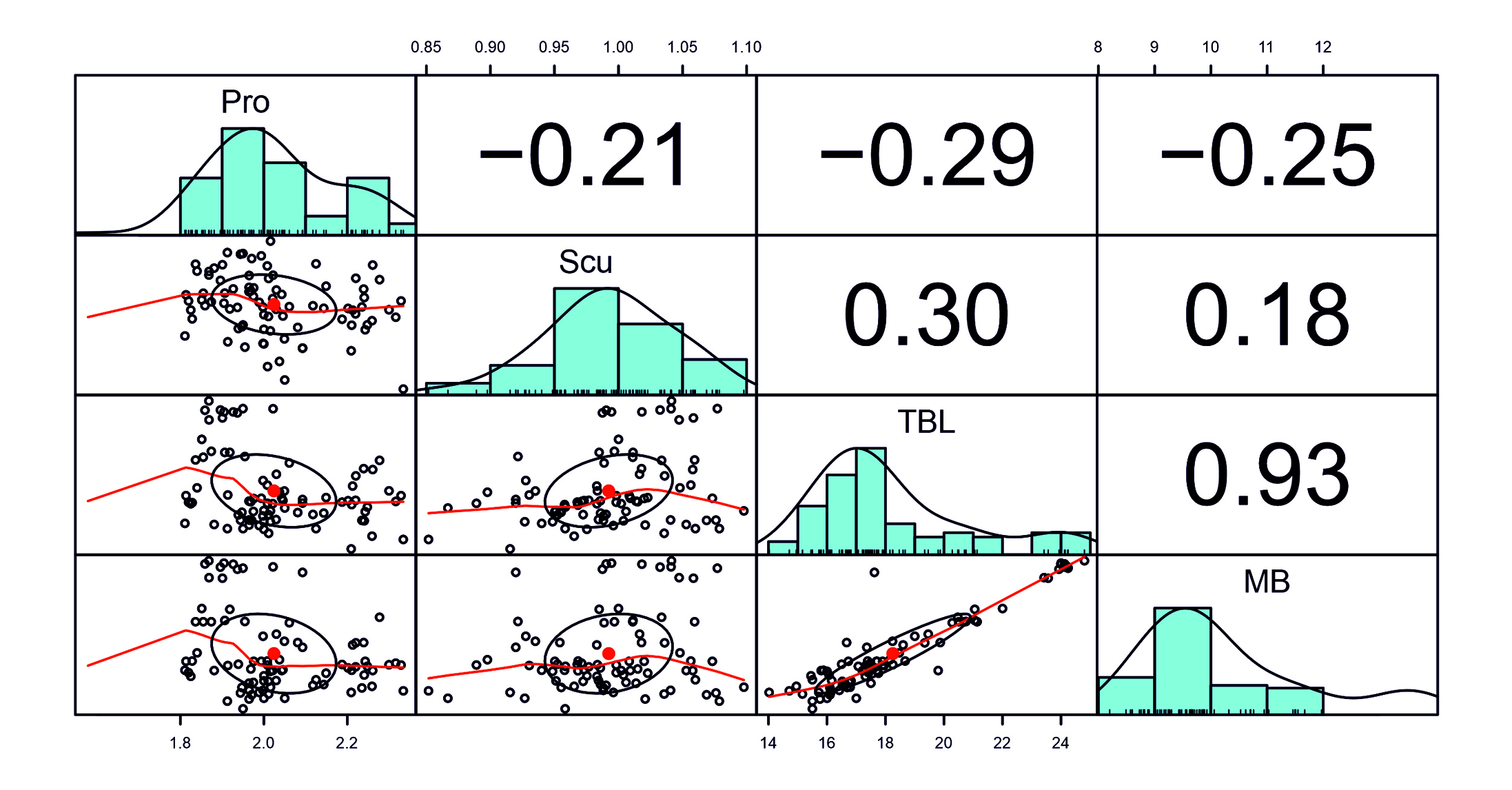

Supplement: S2 Fig — (TIF) [file pone.0298176.s003.tif]
